# Metabolism pathways of arachidonic acids: mechanisms and potential therapeutic targets

**DOI:** 10.1038/s41392-020-00443-w

**Published:** 2021-02-26

**Authors:** Bei Wang, Lujin Wu, Jing Chen, Lingli Dong, Chen Chen, Zheng Wen, Jiong Hu, Ingrid Fleming, Dao Wen Wang

**Affiliations:** 1 Division of Cardiology, Department of Internal Medicine and Gene Therapy Center, Tongji Hospital, Tongji Medical College, Huazhong University of Science and Technology, Wuhan, China; 2 Hubei Key Laboratory of Genetics and Molecular Mechanisms of Cardiological Disorders, Huazhong University of Science and Technology, Hubei Province, Wuhan, China; 3 Department of Rheumatology and Immunology, Tongji Hospital, Tongji Medical College, Huazhong University of Science and Technology, Hubei, Wuhan, China; 4 Institute for Vascular Signalling, Centre for Molecular Medicine, Goethe University, Frankfurt am Main, Germany

**Keywords:** Cancer, Cardiovascular diseases

## Abstract

The arachidonic acid (AA) pathway plays a key role in cardiovascular biology, carcinogenesis, and many inflammatory diseases, such as asthma, arthritis, etc. Esterified AA on the inner surface of the cell membrane is hydrolyzed to its free form by phospholipase A2 (PLA2), which is in turn further metabolized by cyclooxygenases (COXs) and lipoxygenases (LOXs) and cytochrome P450 (CYP) enzymes to a spectrum of bioactive mediators that includes prostanoids, leukotrienes (LTs), epoxyeicosatrienoic acids (EETs), dihydroxyeicosatetraenoic acid (diHETEs), eicosatetraenoic acids (ETEs), and lipoxins (LXs). Many of the latter mediators are considered to be novel preventive and therapeutic targets for cardiovascular diseases (CVD), cancers, and inflammatory diseases. This review sets out to summarize the physiological and pathophysiological importance of the AA metabolizing pathways and outline the molecular mechanisms underlying the actions of AA related to its three main metabolic pathways in CVD and cancer progression will provide valuable insight for developing new therapeutic drugs for CVD and anti-cancer agents such as inhibitors of EETs or 2J2. Thus, we herein present a synopsis of AA metabolism in human health, cardiovascular and cancer biology, and the signaling pathways involved in these processes. To explore the role of the AA metabolism and potential therapies, we also introduce the current newly clinical studies targeting AA metabolisms in the different disease conditions.

## Introduction

**Fig. 1:**
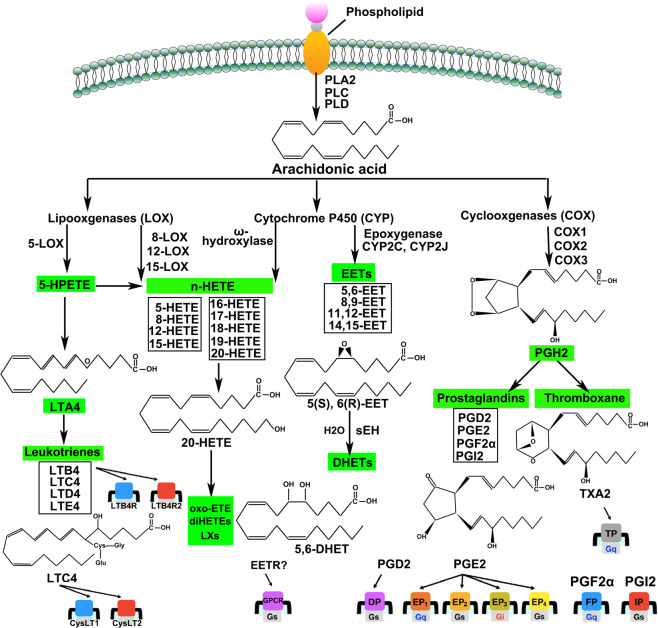
Overview of the arachidonic acid (AA) metabolism pathways. Three major phospholipase enzymes (PLA2, PLC and PLD) are responsible for releasing AA from membrane-bound phospholipids by catalyzing the red arrow indicated covalent bonds, respectively. The PGHSs (COXs) metabolize AA to protanoids, prostacyclin, and thromboxane. The LOXs metabolize AA to leukotrienes and HETEs. The P450 epoxygenases metabolize AA to midchain HETEs and four EET regioisomers. All EETs are then further metabolized to less active dihydroxyeicosatrienoic acids (DHETs) by sEH

The ω-6 polyunsaturated fatty acid (PUFA), arachidonic acid (AA), and its metabolites have attracted a lot of attention in cardiovascular and cancer biology, particularly in relation to inflammatory processes and disease.^1–6^ The importance of AA in biology lies in the fact that it can be metabolized by three distinct enzyme systems, i.e., cyclooxygenases (COXs, also referred to as PGG/H synthases), lipoxygenases (LOXs), and cytochrome P450 (CYP) enzymes (ω-hydroxylases and epoxygenases) to generate an impressive spectrum of biologically active fatty acid mediators (Fig. 1).

The COXs, which generate prostanoids, i.e., prostaglandins (PGs) and thromboxane A_2_ (TXA_2_), were the first enzymes reported to metabolize AA. This requires the release of the lipid from the plasma membrane by phospholipases and subsequent metabolism by the COX enzymes to PGG_2_ and PGH_2_. The latter are then metabolized to PGs by specific PG synthases. There are two distinct COX isoforms; COX-1, which is constitutively expressed in most cells, is the dominant source of prostanoids that subserve housekeeping functions.^7^ COX-2 (also known as PTGS2), on the other hand, is induced by inflammatory stimuli, hormones, and growth factors, is generally assumed to be the more important source of prostanoid formation in inflammation and in proliferative diseases, such as cancer.^7,8^ However, the situation is not black and white as both enzymes contribute to the generation of autoregulatory and homeostatic prostanoids, and both can contribute to prostanoid released during inflammation. Indeed, aspirin and non-steroidal anti-inflammatory drugs (NSAIDs), including inhibitors of COX-2 are effective in the treatment of pain and inflammation.^9,10^ However, the inhibition PGI_2_ production by the endothelium may contribute to the cardiovascular side effects of COX2 inhibitors.^11^ It is thought that inhibition of blood clotting by aspirin can reduce the risk of ischaemic events such as heart attacks and stroke, and prostacyclin analogues are used for the treatment of pulmonary hypertension.^9,12,13^

The LOX pathway was the second eicosanoid and inflammatory pathway to be therapeutically targeted. The enzymes generate leukotrienes (LTs) which were first described in 1979 by Bengt I. Samuelsson who was awarded the Nobel Prize in Physiology or Medicine in 1982.^14^ Arachidonate 5-LOX (or ALOX5) and LT receptor antagonists have been developed for the treatment of asthma and seasonal allergies.^15,16^ These two eicosanoid pathways (COX and LOX) are becoming increasingly important therapeutic targets as novel receptors and metabolites are identified and their roles in many diseases are better defined.

The third AA metabolizing pathway is the cytochrome P450 (CYP) pathway that was first described in 1980. The CYP family of enzymes contains numerous subclasses,^17^ but for the metabolism of AA ω-hydroxylase and epoxygenase activity are the most important. However, numerous CYP enzymes have mixed hyprolase and epoxygenase functions and are able to generate a mixed spectrum of products. The ω-hydroxylase activity of CYP enzymes converts AA to hydroxyeicosatetraenoic acids (HETEs). 20-HETEs is the best-studied metabolite in this context and has been shown to possess pro-inflammatory effects in addition to contributing to vascular function.^18^ The epoxygenase activity of CYP enzymes, such as the CYP2J and 2C families, generates AA epoxides or epoxyeicosatrienoic acids (EETs; 5,6-EET, 8,9-EET, 11,12-EET, and 14,15-EET). Bioactive EETs are produced in the liver with biologically relevant amounts also being detected in the vascularure as well as in cardiomyocytes. The EETs are mainly metabolized by soluble epoxide hydrolase (sEH) to the corresponding diols or dihydroxyeicosatrienoic acids (DHET).^19,20^ AA diols were initially thought to be less active than the epoxides, but it is now clear that the epoxide and diols may even exert antagonistic actions in some conditions. As the EETs are reported to elicit vasodilatation, this pathway and its metabolites are currently being targeted for the treatment of cardiovascular diseases (CVDs) including hypertension, heart failure (HF), and stroke.^21,22^ In addition, CYP-derived EETs also regulate some cellular processes of carcinogenesis and progression, including cell proliferation, survival, angiogenesis, invasion, and metastasis. CYP-derived EETs can also promote progenitor cell differentiation, proliferation, and migration, in addition to influencing capillary formation inflammation and apoptosis in endothelial cells. In this way CYP-derived AA metabolites can contribute to tumor growth, progression, and metastasis.^23^

In this Review, we focus on recent insights into the roles of AA metabolism from molecular mechanisms to clinical studies, particularly in CVD, cancer biology and inflammatory diseases.

## Overview of AA metabolism

### The COX pathway

As stated above, the term COX refers to enzymes also known as prostaglandin G/H synthases (PGHS), which metabolize AA to PGH_2_ and PHG_2_. These PGs are substrates for a series of downstream enzymes that generate specific PGs i.e. PGE2, PGI2, PGD2, PGF2, and TXA2.^24–26^ The major difference between the 2 COX enzymes is that while COX-1 is more or less ubiquitously and constitutively expressed, COX-2 is an inducible enzyme,^26–28^ albeit with some important exceptions.^29,30^ There are preferences in the coupling between COX and downstream synthases as COX-1 couples preferentially, but not exclusively, with thromboxane synthase, PGF synthase, and the cytosolic (c) PGE synthase (PGES) isozymes. COX-2, on the other hands, more frequently feeds PGG2/H2 to the prostaglandin I synthase (PGIS) and the microsomal (m) PGES isozymes, both of which are often coinduced with COX-2 by cytokines and tumor promoters.^31–34^ The profile of prostanoid production is determined by the differential expression of these enzymes within cells present at sites of inflammation. For example, mast cells predominantly generate PGD2, whereas macrophages produce PGE2 and TXA2.^35^ In addition, alterations in the profile of prostanoid synthesis can occur on cellular activation. An additional COX enzyme, i.e., COX-3, a splice variant of COX-1^36^ that also produced PGH2 has been identified and its expression is reportedly higher in microvessels of the brain and heart than in larger conduit arteries.^37,38^

PGs exert their effects by activating membrane-localized G protein-coupled receptors and the prostanoid receptor subfamily is composed of 8 members; the PGD receptor (DP1), the PGF receptor (FP); the PGI receptor (IP), the thromboxane receptor (TP), and 4 subtypes of E prostanoid receptor (EP1-4).^39^ Alternative splicing complicates the situation further and is responsible for two additional isoforms of the human TP (TP_α_, TP_β_) and FP (FP_A_, FP_B_) receptors as well as eight variants of EP3 which differ only in their C-terminal tails.^40^ In addition, there is a distinct G protein-coupled receptor, i.e., chemoattractant receptor-homologous molecule (CRTH2 or DP2) that is expressed on T helper 2 cells that belongs to the family of chemokine receptors but can be activated by PGD2.^40,41^ Prostanoid receptors couple to a range of intracellular signaling pathways that mediate the effects of receptor activation on cell function. For example, the EP2, EP4, IP, and DP1 receptors activate adenylyl cyclase via Gs, to increase intracellular cAMP whereas EP1 and FP activation couples to phosphatidylinositol metabolism via Gq, leading to the formation of inositol trisphosphate with mobilization of intracellular free calcium.^42,43^

### The LOX pathway

The LOX enzymes insert molecular oxygen in AA and depending on its position, 4 hydroperoxyeicosatetraenoic acids (HPETEs; 5-, 8-, 12-, and 15-HPETE) are formed by the corresponding LOX enzymes, i.e., 5-LOX, 8-LOX, 12-LOX, and 15-LOX. The HPETEs are then reduced into monohydroxy eicosatetraenoic acids (HETEs) by peroxidases, or converted to biologically active compounds such as LTs, lipoxins (LXs), and hepoxilins.

Perhaps the best-studied LOX enzyme is 5-LOX, which inserts oxygen into AA at the C-5 position to generate 5-HPETE and subsequently LTA4, the precursor of the LTs (LTB4, LTC4, LTD4 and LTE4).^44–46^ Although 5-LOX was originally purified as a cytosolic protein, it was later shown to translocate to the nuclear envelope after phosphorylation.^47,48^ It is now accepted that the nuclear membrane is the major site for the production of LTs. 5-HPETE is further hydrolyzed by LTA4 hydrolase to generate LTB4.^48,49^ For its catalytic activity 5-LOX requires a 5-LOX-activating protein (FLAP),^50,51^ a membrane-spanning protein with three transmembrane domains belonging to the “membrane-associated proteins in eicosanoid and glutathione metabolism (MAPEG)” family that includes LTC4 synthase and microsomal PGE2 synthase.^15,48,52^ The precise role of FLAP in 5-LOX reactions remains to be fully elucidated but it is thought that FLAP presents AA to 5-LOX and/or functions as a scaffold for 5-LOX.^53^ A large body of work now documents the role of 5-LOX-generated products in the pathogenesis and progression of CVD,^54^ particularly atherosclerosis, MI, stroke, aortic aneurysms, and intimal hyperplasia. 5-LOX-derived mediators in particular focus are oxo-ETEs generated from HETEs by the microsomal dehydrogenase in polymorphonuclear leukocytes (PMNLs), which now seems to be a strong eosinophil chemoattractant.^55^ Also, LTs are now recognized as a crucial component of vascular inflammation.^56^ These are generated by is a bi-functional enzyme, i.e., the LTA4 hydrolase—a cytosolic protein that has both LTA4 hydrolase and zinc-dependent peptidase activities. Although the biological role of the LTA4 hydrolase as a peptidase is unknown, it limits pulmonary inflammation by degrading the chemotactic peptide PGP (proline-glycine-proline).^57^ Thus, in inflammation the LTA4 hydrolase generates a chemotactic lipid mediator, i.e., LTB4, at the same time as degrading a chemotactic peptide, i.e., PGP. Two major pathways of LTB4 inactivation are known, and responsible enzymes have been identified. Granulocytes and hepatocytes inactivate LTB4 through the ω-oxidation pathway^58^ in which C-20 of LTB4 is oxidized by CYP enzymes; CYP4F3 in granulocytes and CYP4F1 and 2 in hepatocytes.^59^ In other tissues, LTB4 is inactivated by conversion into 12-keto-LTB4 by the LTB4 12-hydroxydehydrogenase,^48,60^ which is also involved in the inactivation of various eicosanoids including PG^48^ and LXA4.^61^ As far as signaling is concerned, LTC4 exerts its actions on smooth muscle contractions through CysLT1 and CysLT2 receptors. LTB4, on the other hand, acts via LTB4R (BLT1) and LTB4R2 (BLT2) receptors.^62^

In addition to their ability to generate HETEs via a similar process as described above for 5-LOX, 12-LOX and 15-LOX also generate LXs, oxo-ETEs, and dihydroxyeicosatetraenoic acids (diHETEs).^63^ For example, 12-LOX can convert 5(S)-HETE to 5(S),12(S)-diHETE as well to 14(R),15(S)-diHETE in the, which ultimately contribute to the generation of extra-platelet LTA4.^64,65^ The formation of LXs involves 5-LOX in neutrophils and 12-LOX in platelets. In neutrophils, 5-LOX generates LTA4, which is then transferred to platelets where 12-LOX subsequently generates either LXA4 or LXB4.^66,67^ There are two isoforms of 15-LOX in mammalian cells: 15-LOX-1 and 15-LOX-2. 15-LOX-1 is encoded by the arachidonate 15-lipoxygenase (ALOX15) gene, and the functional enzyme metabolizes AA to LXA4, LXB4, and 15-oxo-ETEs. 15-LOX-2, on the other hand, generates 15-oxo-ETE and 8S-HETE.^68,69^ The efficiency of 15-LOX-1 is 20 times higher than that of 12-LOX,^66^ thus when 15-diHPETE is provided as substrate, the primary product catalyzed by 12-LOX and 15-LOX-1 is LXB4.

### The CYP pathway

CYP genes encode a super-family of mixed-function monooxygenases, which includes more than 6000 individual enzymes (http://drnelson.uthsc.edu/CytochromeP450.html).^70^ The best-known role of the CYP pathway is the metabolism of lipophilic xenobiotics, including drugs and chemical carcinogens, as well as endogenous compounds such as steroids, fat-soluble vitamins, fatty acids, and biogenic amines. CYP expression and activity are under the control of hormones, growth factors, and transcription factors. Indeed, different CYP subfamilies can display complex tissue- and development-specific expression patterns. Despite this, CYP2C and CYP2J enzymes can be detected in hepatocytes, cardiomyocytes, vascular endothelial cells, smooth muscle cells, and in some epithelial cells, autonomic ganglion cells, hepatocytes, nerve cells, and islet cells.^71^ To-date perhaps the most impressive links with biological activity are for EETs in liver, kidney, heart, and endothelial cells.^71^ Importantly, many genetic and environmental factors alter CYP expression resulting in significant changes in the production or removal of bioactive products.

As far as the cardiovascular system is concerned CYP enzymes are important as they generate by ω-hydroxylated HETEs (6-, 17-, 18-, 19-, and 20-HETE). Perhaps the best studied to these is 20-HETE, which has been linked with vasoconstriction and the regulation of myogenic tone.^18^ The AA epoxides or EETs, i.e., 5,6-, 8,9-, 11,12- and 14,15-EET, have vasodilatory, cardioprotective, and anti-inflammatory activities and can modulate vascular smooth muscle migration, an important event in vascular remodeling and atherosclerosis. Each of the 4 EET regioisomers has stereoisomers, e.g., 11,12-EET exists as 11(R),12(S)-EET and 11(S),12(R)-EET, and the different stereoisomers can elicit distinct actions.^72^ The intracellular levels of the EETs are tightly regulated by the activity of the sEH, which generates the equivalent DHETs. The latter has traditionally been considered to be less active than their parent EETs. Relevant human CYP enzymes contributing to the formation of EETs and their distribution are listed in Table 1. Although EETs exhibit some similarities in biological functions, there are differences in their actions to some extent. For example, EETs were found to be a slightly stronger pro-angiogenic factor than other in vivo and in vitro.^73,74^ CYP-derived EETs are probably best studied with respect to their hyperpolarizing properties as EETs are endothelium-derived hyperpolarizing factors (EDHF) in some organs (particularly in the heart) and thus contribute to the regulation of vascular function.^19^ It is also now clear that CYP-derived EETs also protect the heart against acute ischemia-reperfusion injury and chronic non-ischemic cardiomyopathy and hypertension.

**Table 1:** Relevant human CYP epoxygenases contributing to the formation of EETs and their distribution

CYP	% Epoxidation	AA epoxidation metabolic rate (min^−1^)	Liver		Heart		Aorta		Brain		Intestinal		Lung		Kidney	
			Pro	RNA	Pro	RNA	Pro	RNA	Pro	RNA	Pro	RNA	Pro	RNA	Pro	RNA
CYP1A2	60	1.8(50 μM)	15–52	+++	det					det				det	det	
CYP3A4	35	1.5(50 μM)	40–155	+++							8.8–150			+		+
CYP3A5	nd	nd	1–68	++							4.9–25					
CYP2C8	70	0.16(10 μM)	24–64		0.2	+++	nd	++	det				det			+++
CYP2C9	75	0.36(10 μM)	73–120		nd	+++	1.3	+++	det		2.9–27		det			+++
CYP2C18	–	0.07(10 μM)	<2.5													
CYP2C19	75	0.6(10 μM)	14–30								0.6–3.9					
CYP2J2	100	0.1(50 μM)	2		0.05–0.4	+++	0.06	+	det	+	0.2–3.1	+		+	det	+++
sEH	na	na	det		det		det		det	+	det	++	det	++	det	

Abundance as approximate values in pmol/mg for protein; mRNA level: + low, ++ moderate, +++ high

nd not detected, det detected, na not application

References:^515–519^

## AA metabolites in CVD

CVD remains a major cause of disability and death in both Western societies and developing nations. As age and co-morbidities, such as obesity and diabetes, become more prevalent in a population both the human health cost and economic burden of these conditions keep increasing. There is compelling evidence of a role for some AA metabolites generated by COX, LOX and CYP enzymes in the development and progression of CVD.^75–77^

### Role of COX enzymes and their products in CVD

#### COXs and CVD

The COX pathway is one of the major treatment targets in atherosclerotic and ischemic heart disease because it affects major pathophysiological features of these diseases, including platelet aggregation, vessel wall tension, and inflammatory processes in atherosclerotic lesions.^12^ The anti-inflammatory and anti-thrombotic features of aspirin, the only known irreversible inhibitor of COX-1, are primarily related to the suppression of PG and TXA2 synthesis.^78,79^ Meta-analyses of randomized trials show that the use of aspirin lowers the risk of cardiovascular events by 15% and myocardial infarction (MI) by as much as 30%.^80^ Beyond effects on platelets, it seems that the COX-1/TXA2 pathway contributes to vascular hypercontractility in atherosclerotic ApoE-deficient mice, targeting this pathway pharmacologically improves endothelial function.^81^ Aspirin is the only known nonsteroidial anti-inflammatory drug (NSAID) with cardiovascular protective effects but despite its widespread and long-term use, some aspects of aspirin treatment warrant further investigation; such as the interaction between body weight and the effectiveness of aspirin to prevent cardiovascular events.^76^ COX-2 expression increases with inflammation and although COX-2 inhibitors preserve left ventricular function and dimensions in murine models of MI, these compounds have been found to increase cardiovascular risk in multiple clinical studies. For example, a retrospective cohort study including over 300,000 individuals suggested that the use of two highly selective COX-2 inhibitors; valdecoxib and rofecoxib, was associated with a higher risk of stroke.^82^ Also, rofecoxib and etoricoxib increased blood pressure, whereas other members of this class of compound, i.e., celecoxib, lumiracoxib, and valdecoxib appeared to have little effect on blood pressure.^83^ Another retrospective cohort study of over 2000 individuals aged over 65 also indicated a higher combined risk of recurrent congestive HF and mortality in patients prescribed with refecoxib rather than celecoxib.^84^ The reason for these negative cardiovascular effects seems to be related to inhibition of PGI2 production by the COX-2 expressed by the vascular endothelium exposed to “atheroprotective” laminar flow.^85,86^ The potent vasoconstrictor 20-HETE is also affected by COX-2 inhibition as it is at least partially inactivated by a COX-2-dependent metabolic step.^75,87^ Combined therapeutic approaches may get around some of these issues and a new class of drugs that blocks both the COX-1/2 and 5-LOX pathways, may prove to be an effective treatment option for cancer, inflammatory and CVDs, with fewer side effects.^88^ The compound currently in the most advanced phase of clinical development (phase III) is licofelone, previously known as ML3000.^89^ Licofelone, characterized as a FLAP inhibitor and also has a weak effect on microsomal prostaglandin E synthase-1 (mPGES-1), developed by Merckle and the partners Alfa Wassermann and Lacer, has reached clinical phase III for the treatment of knee osteoarthritis^90^ with several studies successfully completed. Compared with other nonsteroidal anti-inflammatory drugs (NSAIDs), licofelone showed improved gastric tolerability and lower incidences of ulcers in healthy volunteers.^91^

#### COX products and ischemic cardiomyopathy

A more detailed analysis of the role of prostanoids in the pathogenesis of CVD has been possible with the generation of mice lacking either enzyme involved in prostanoid biosynthesis of prostanoid receptors.^12,92,93^ Such studies have revealed important and novel roles for prostanoids in the development of acute myocardial infarction (AMI), cardiac hypertrophy, hypertension, atherosclerosis, and vascular remodeling.

PGI2 and TXA2 are the major prostanoids affecting the cardiovascular system and are mainly produced by vascular endothelial cells and platelets.^94^ Importantly, these compounds are often functional antagonists, i.e., they exert directly opposing effects on a given cell or tissue. This means that the balance in their production is crucial for the maintenance of vascular homeostasis. A shift away from PGI2 towards TXA2 can contribute to the development of various thrombotic diseases.^95^ Both mediators can also be produced by cardiomyocytes, and their synthesis increased significantly during cardiac ischemia and reperfusion,^94,96^ suggesting a potential contribution to reperfusion injury. Certainly, PGI2 and its analogues attenuate cardiac reperfusion injury in vivo.^97,98^ Similarly, TX synthase inhibitors and/or TP antagonists reduce myocardial infarct size in animal studies.^99,100^

There is evidence for a role of other prostanoids in CVD and PGE2 production also increases during AMI.^101,102^ What contribution the endogeneously generated PGE2 makes to tissue defence or disease progression has, however, not been determined. More is known about its receptors and even though the expression levels of each EP subtype varied among the species studied, high levels of the EP4 mRNA have been reported in the hearts of several species, including humans.^8,103^ Using EP4^−/−^ mice it was possible to demonstrate that mice lacking EP4 developed larger infarcts in a model of ischemia and reperfusion. Moreover, isolated perfused hearts (Langendorff preparation) from EP4^−/−^ mice demonstrated more pronounced functional and biochemical derangements in response to ischemia and reperfusion than hearts from wild-type mice.^103^ EP4 agonists have also been developed and despite the fact that one such compound elicited only weak effects in cardiomyocytes, it markedly increased cAMP concentrations in noncardiomyocytes.^103^ A second EP4 agonist, significantly reduced infarct size in wild-type mice when administered 1 h prior to coronary occlusion. These results indicate that PGE2 produced endogenously during ischemia or reperfusion can protect the heart from injury.^103^ Less is known about EP3 receptors but several studies indicate that EP3 agonists also protect the heart from injury by facilitating the opening of K_ATP_ channel, also the cardio-äspecific overexpression of EP3 attenuated myocardial ischemia-reperfusion injury.^104–106^

#### COXs-derived metabolites and cardiac hypertrophy

The role of prostanoids in cardiac hypertrophy has been examined using animal models of pressure overload- and angiotensin II (Ang II)-infusion.^107,108^ One example is PGI2 as it (and its analogues) can inhibit the Ang II-induced hypertrophy of cultured cardiomyocytes,^107^ as well as the proliferation and synthesis of collagen by cultured cardiac fibroblasts.^109,110^ In a more pathophysiologically relevant situation, the PGI2-IP system attenuated the development of pressure overload-induced cardiac hypertrophy by inhibiting both cardiomyocyte hypertrophy and cardiac fibrosis. Specially, the hypertrophic effect of PGF2α on cultured rat cardiomyocytes was not observed in mice due to defective FP signaling.^111^ Somewhat intriguingly, it seems that PGE2-EP3 is necessary to maintain the normal growth and development of the heart.^112^ Indeed, the cardiomyocyte-specific deletion of EP3 induces eccentric cardiac hypertrophy and cardiac fibrosis in 16–18-week-old mice, supposedly by inactivating the mitogen-activated protein kinase/extracellular signal-regulated kinase (MAPK/ERK) pathway and affecting matrix metal proteinase 2 (MMP-2) expression. Studies on EP4-mediated responses are hampered by the fact that most EP4^−/−^ neonates become pale and lethargic within 24 h of birth and die within 72 h. This phenomenon has been attributed to a failure of the ductus arteriosus to close, and in situ hybridization study showed that EP4 mRNA is strongly expressed in the ductus, suggesting that the receptor plays a role in the regulation of the patency of this vessel.^113^ Such results also indicate that the normal function of the EP4 receptor is essential for the rapid adaptation of the circulatory system in neonates.^113^

#### COXs-derived metabolites and hypertension

Genetic disruption of the EP1 receptor is reported to blunt the acute pressor response to Ang II as well as to reduce chronic Ang II-driven hypertension.^114^ Also, oral administration of an EP1 receptor antagonist reduced blood pressure in spontaneously hypertensive rats. EP2^−/−^ mice, on the other hand, develop normally but produce small litters and have slightly elevated baseline systolic blood pressures. These animals lacked the characteristic hypotensive response to the intravenous infusion of PGE2, which was in fact converted to hypertension. Such data demonstrate that the EP2 receptor mediates arterial dilatation, salt-sensitive hypertension, and also plays an essential part in female fertility.^115^ However, PGI2-IP and TXA2-TP system has been reported to be resistant to renovascular hypertension or Ang II-induced hypertension.^108,116^ In addition, the endothelial expression of PGD synthases, which is responsible for PGD2 synthesis from PGH2, can be upregulated in response to higher shear stress in the circulation.^117^ Genetic deletion of lipocalin-type PGD synthases in mice triggers hypertension and thrombogenesis.^92^

### Role of LOX enzymes and their products in CVD

During the early phase of inflammation, AA is predominantly metabolized via 5-LOX which generates pro-inflammatory LTs including LTB4. In the later stages of inflammation moving towards resolution PGs, such as PGE2, increase 15-LOX expression which redirects the flow of substrate away from LTB4 synthesis to 15-LOX and the production of LXA4 production. Notably, in vivo levels of LXA4 are decreased in patients with peripheral and coronary atherosclerosis,^118^ and the overexpression of 12-LOX and 15-LOX in macrophages of atherosclerotic ApoE-deficient mice increase LXA4 production and hamper atherosclerotic lesion development. This atheroprotective effect of LXA4 has been linked to its anti-inflammatory capacity, as it impairs the production of various pro-inflammatory cytokines, stops neutrophil chemotaxis, and induces pro-resolving macrophages functions.^78,119,120^ Interestingly, aspirin enhances LXA4 production ensued by alleviation of atherosclerotic lesions in ApoE deficient mice.^121^ Efferocytosis, especially the clearance of polymorphonuclear cells (PMNs) and foam cells, is of major importance for the resolution of inflammation, and its impairment leads to prolongation and progression of inflammatory situations including atherosclerosis. LXs produced by LOX enzymes contribute to the process of efferocytosis.^122^ Moreover, the protective role of most widely used statin therapies in CVD seems to be (at least partly) attributable to LXA4. Indeed, atorvastatin^123^ and simvastatin^124^ can increase the myocardial content of LXA4 and 15(R)-epi-lipoxin-A4 (15-epi-LXA4), both AA products with strong anti-inflammatory properties.^125^ Despite this, the atheroprotective functions of 12/15-LOX-derived metabolites remain controversial, as 12/15-LOX-ApoE-double-deficient mice were found to be less prone to atherogenesis than ApoE^−/−^ littermates with fully functional 12/15-LOX enzymes.^126^

In contrast to the mainly atheroprotective roles attributed to the LXs, LTs promote the progression of hyperlipidemia-dependent vascular disease and are associated with atherogenesis, CVD, MI, and stroke.^15,127,128^ In addition, LTB4 and CysLTs are likely to contribute to the pathophysiology of atherosclerosis and myocardial dysfunction. Accordingly, enhanced activity of the 5-LOX pathway was detected in atherosclerotic lesions,^129^ and the quantity of 5-LOX positive cells correlated with atherosclerotic lesion progression and plaque stability.^78,129^ Fitting with this, blocking LTB4 receptors protects against the development of atherosclerosis in ApoE^−/−^ mice,^130^ and the endothelial overexpression of endothelial cysteinyl leukotriene 2 receptor (CYSLTR2) increase vascular permeability, myocardial ischemia/reperfusion damage, and cardiomyocyte apoptosis in peri-infarct areas.^78,131,132^ LTB4 also fosters the recruitment of neutrophils to atherosclerotic plaques and contributes to plaque destabilization.^133^ In line with the pro-atherogenic effects of LTs, they are implicated in myocardial ischemia and reperfusion injury. Accordingly, CYSLTR2 expression within the heart and vasculature is induced by ischemia/reperfusion injury.^134^ The interaction of LTs with CYSLTR2 increases vascular permeability and amplifies the extent of the myocardial injury, and high levels of CYSLTR2 expression in the heart and vessels have been linked to a detrimental outcome in murine ischemia/reperfusion models.^78,134^ In line with this, pharmacological blockade of LTBR4 reduces infarct size in a murine model of myocardial ischemia/reperfusion injury,^135^ and the CYSLTR antagonist; montelukast, which is mainly used in the treatment of asthma and allergic rhinitis, was recently evaluated for its possible cardio-protective effects. Interestingly, both animal models and clinical trials demonstrated a preventive role of montelukast against the development of atherosclerosis and suggested a cardioprotective function.^136–138^

### Roles of CYP enzymes and their products in CVD

#### CYP-derived EETs and heart functions

**Fig. 2:**
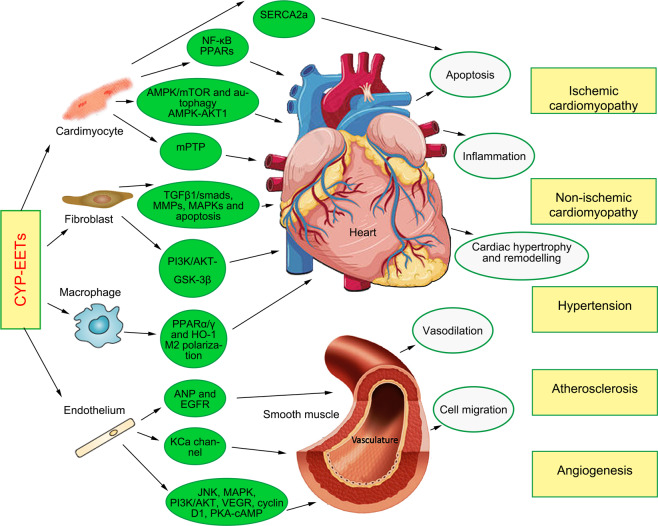
The main biological functions of CYP-EETs on the cardiovascular system and the main corresponding cellular signaling pathways. CYP epoxygenase metabolites of AA, EETs, act as lipid mediators eliciting numerous biological responses and impacting both vascular and cardiac function, including anti-apoptosis, anti-inflammation, vasodilation, inducing angiogenesis, anti-hypertension and aginst ischemic cardiomyopathy or non-ischemic cardiomyopathy

It is well established that the epoxides of AA generated by CYP enzymes possess biological activity and affect a wide spectrum of cellular and tissue responses as well as having effects on the cardiovascular system. Perhaps most work on the EETs has been performed on vessels and vascular cells and less is known about the actions of cardiac-specific CYP-derived EETs in heart physiology and pathophysiology (Fig. 2), compared with the cardiac expression of CYP subfamilies identified in mammalian species including CYP1A, CYP1B, CYP2A, CYP2B, CYP2D, CYP2E, CYP2J, CYP2R, CYP2S, CYP2U, CYP4A, CYP4B, CYP4F, and CYP11B.^139^

##### CYP-derived EETs and ischemic cardiomyopathy

Ischemic cardiomyopathy is defined as CVD resulting from a period of low oxygen flow to the heart.^140^ This could be due to a blockage resulting in limited blood flow, and consequently oxygen, to the heart. Reduced oxygen levels lead to a wide range of consequences for heart activity and morphology that are detrimental to proper function and homeostasis.^140^ Overall, CYP-derived EETs in the heart has been shown to improve the outcomes of ischemia and/or ischemia/reperfusion injuries.^141,142^ This is relevant inasmuch as the expression of many CYP enzymes is increased by hypoxia,^143^ while that of the sEH is decreased^144^—conditions that would favor EET stability and bioavailability.

Myocardial ischemia/reperfusion (IR) injury occurs when the coronary flow is obstructed, resulting in widespread damage and remodeling of the heart. MI is characterized by extensive fibrosis, remodeling, inflammation, and myocardial apoptosis that eventually progresses to HF and mortality. The immune system plays an important role in the physiopathology of MI, the increased number of circulating inflammatory leukocytes can lead to more cells accumulating in the myocardium.^145^ Upon accumulating in the heart, neutrophils, and monocytes participate actively in the inflammatory cascade. Neutrophils do not persist in the infarcted myocardium for very long; their numbers decrease after 3 days, and they almost entirely disappear after 7 days. However, neutrophils have been shown to improve cardiac healing by promoting macrophage polarization towards a reparative phenotype through the release of neutrophil gelatinase-associated lipocalin.^146^ Thus, although antibody-mediated depletion of neutrophils does not affect infarct size, it does worsen cardiac function and HF, and it also increases cardiac fibrosis.^146^ In contrast to neutrophils, monocytes continue to accumulate in the ischaemic heart and differentiate to cardiac macrophages for several days. The bone marrow maintains leukocyte production but also expels HSPCs, which accumulate in extramedullary sites such as the spleen. Therein, these cells begin to produce monocytes and neutrophils via a process known as extramedullary hematopoiesis, which increases the number of immune cells that can be recruited to the heart.^147^ Over the course of several days, the inflammatory phase gives way to a reparative phase,^148^ which is dominated by the disappearance of neutrophils and the appearance of Ly6C^low^ macrophages. The transition from inflammation to repair is driven by decreased production of inflammatory cytokines, growth factors, and chemokines.

The common method of inducing MI in vivo is through left anterior descending artery (LAD) occlusion.^149,150^ This results in a clear and defined infarct region and mimics much of the injury and functional deficits are seen post-MI in humans. Ex vivo models include isolated Langendorff or working heart models to induce IR injury. In vitro, hypoxia/reoxygenation models are typical, although not exactly equivalent to ischemic injury since lack of blood flow in vivo comes with other consequences.^139^

Models that increase EET bioavailability in mice include the cardiomyocyte-specific overexpression of the human CYP2J2 in C57/BL6 mice, an intervention that improved left ventricular recovery after ischemia and reperfusion.^151^ Moreover, EET augmentation (mainly 11,12-EET)^151^ has shown beneficial effects on the chronic effects of such injury. Specifically, preventing the metabolism of EETs by the sEH improves the murine myocardial ejection fraction following LAD ligation and has also been associated with improved myocardial perfusion.^152^ Similarly, administering EETs for as long as a week following infarction is associated with a reduction in fibrosis. The potentially protective actions of the EETs involve the inhibition of apoptosis, the promotion of pro-survival signaling as well as preserved mitochondrial structure and function. Recently, the endothelial cell-specific overexpression of CYP2J2 was found to improve cardiac function by promoting angiogenesis via Jagged1/Notch1 signaling in a mouse model of LAD ligation. This fits with earlier in vitro studies showing that 11,12-EET and also other EETs induces more robust tube formation and markedly increased vascular endothelial growth factor (VEGF)-A^74^ and basic fibroblast growth factor (bFGF) expression in hypoxia and normoxia,^142^ indicating that CYP2J2 in endothelium also contributed to cardioprotection. Moreover, isolated mouse hearts treated directly with EETs or dual-acting compounds possessing EET mimetic and sEH inhibitory properties had reduced infarct size and preserved left ventricular developed pressure (LVDP) compared to controls.^142,153^ There is evidence to indicate that the protective effect of CYP-EETs on ischemia-reperfusion injury may be age-dependent as the cardioprotective effect of CYP2J2 overexpression declined significantly in old (11–13 months) mice.^154^ While the molecular events active by the EETs that underlie such protective mechanisms are unknown, results from rat, mouse, and canine models have provided consistent evidence to suggest that the activation of the K_ATP_ channels and phosphatidylinositol-3 kinase (PI3K) signaling are involved in EET-associated cardioprotection.^155,156^ PI3Ks are members of a family of lipid kinases that phosphorylate the 3′-hydroxyl group of phosphatidylinositol (PIP) and PIP2 at the third position, to form PIP2 and PIP3, which activate downstream kinases such as AKT and glycogen synthase kinase 3 (GSK-3β), which during ischemia-reperfusion injury results in reduced cell death and infarct size.^157^

##### CYP-derived EETs in non-ischemic cardiomyopathy

In broad terms, non-ischemic cardiomyopathy is myocardial injury leading to arrhythmia, ventricular dysfunction, and HF that is not directly associated with AMI.^158^ Causes of NICM are complicated and varied including drug toxicity, genetic predisposition, infection, haemodynamic pathology, and immunologic abnormalities.^158^ Several models are often employed to induce NICM in in vivo, such as transverse aortic constriction (TAC), a surgical model used to stimulate pressure-induced HF, or infusion of Ang II or isoprenaline to induce cardiac hypertrophy and failure.^139,159^ EETs have demonstrated significant cardioprotective effects in models of non-ischemic cardiomyopathy unrelated to their use as anti-hypertensive agents.^160,161^ In fact, CYP-derived EETs and sEH inhibitors may represent a promising therapeutic approach for combating detrimental cardiac remodeling and decline of cardiac function, which is a hallmark of NICM. For example, the cardiomyocyte-specific overexpression of CYP2J2 to increase epoxide levels attenuated Ang II-induced cardiac hypertrophy and remodeling via a mechanism dependent on AMPKα2 and a subsequent increase in atrial natriuretic polypeptide (ANP),^161^ which acts as a vasodilator as well as an inhibitor of fibrosis and renin/aldosterone secretion.^162^ Importantly, ANP mRNA levels were found to be upregulated 6–14 fold in the myocardium following the AAV-mediated overexpression of CYP2J2 in spontaneously hypertensive rats, an effect that was associated with increased ANP expression in the myocardium and elevated plasma levels of the peptide.^163^ The relationships described were causative as 11,12-EET stimulated the γ1 domain of the AMP-activated protein kinase (AMPK) α2β2γ1 to bind directly with the protein kinase domain of AKT1, thus accelerating its translocation to the nucleus resulting in increased expression of ANP and abrogation of cardiac hypertrophy.^161^ In addition, cardiomyocyte-specific expression of CYP2J2 or treatment with EETs protects against cardiac remodeling.^160^ In Ang II-infused mice overexpressing CYP2J2 specifically in cardiomyocytes, cardioprotection was linked with the activation of peroxisome proliferator-activated receptor (PPAR)-γ, reduced oxidative stress, a decrease in nuclear factor (NF)-κB p65 nuclear translocation, and inhibition of transforming growth factor (TGF)-β1/Smad pathway.^160^ Similarly, when ISO or Ang II were used to induce cardiac fibrosis, hypertrophy, and dysfunction, beneficial consequences of CYP2J2 overexpression were linked to attenuated NF-κB activation.^164^ In in vitro experiments, 11,12-EET attenuated cardiomyocyte hypertrophy and the expression of remodeling-related proteins, i.e., collagen I, TGF-β1, tissue inhibitor of matrix metallopeptidase-1 (TIMP1), by similar oxidative stress, NF-κB, PPAR-γ pathway. In an Ang II-induced model of non-ischemic cardiomyopathy, the inhibitory effects of CYP2J2 on cardiac fibrosis were associated with reduced activation of the G12 family Gα proteins (Gα12/13),^165^ which play a pivotal role in regulating the phenotype of cardiac fibroblasts.^166^ The latter studies fit well with numerous in vitro and in vivo reports linking the anti-inflammatory properties of EETs with inhibition of the IκBα kinase (IKK)-NF-κB cascade activated by tumor necrosis factor-α.^167–169^ Additional mechanisms attributed to EETs in models of agonist-induced HF has linked CYP2J2 and EETs with reduced endoplasmic reticulum (ER) stress and apoptosis cumulating in improved systolic and diastolic function.^170^ While EETs can directly affect cardiomyocytes, it is clear that other cardiac cell types are also targeted by EETs, e.g., 14,15-EET treatment suppressed the cardiac inflammatory response, at least in part by preventing macrophages activation.^164^ Interesting data investigating the protective response of EETs toward LPS-induced cardiac dysfunction also revealed decreased NF-κB activation and the upregulation on PPARα/γ and hemeoxygenase-1 (HO-1) to promote the pre-resolution macrophage phenotype.^171^ In an experimental approach to increase the biosynthesis of endogenous EETs, overexpression of CYP2J2 in both cell culture and mouse models, attenuated cardiac dysfunction arising from systemic inflammation caused by TNF-α administration.^169^

Preventing the metabolism of EETs to DHETs by inhibiting the sEH prevented AngII-induced cardiac hypertrophy, in fact, there is a lot of evidence linking AngII with increased sEH expression.^172^ In a TAC mouse model, beneficial effects of sEH inhibition were noted in the prevention of ventricular arrhythmias that occur in association with cardiac hypertrophy.^173^ Similarly, sEH-deficient mice that underwent either TAC- or Ang II-induced hypertrophy demonstrated preserved cardiac function compared to controls. Importantly, the sEH^−/−^ mice displayed a stable sinus rhythm with prolonged cardiac repolarization, indicating a protective effect of gene ablation on cardiac arrhythmias.^174^ Comparable studies in mice with the cardiomyocyte-specific over-expression of CYP2J2 and subjected to TAC or ISO infusion revealed that enhanced cardiac EET biosynthesis is protective against electrical remodeling, ventricular tachyarrhythmia, and atrial fibrillation associated with cardiac hypertrophy.^175^ The increased survival rate observed in CYP2J2 transgenic mice is attributed to better cardiac electrical stability as only moderate improvements were observed in pump function or hypertrophy.^175^ Other studies using sEH inhibitors as an approach to increase the bioavailability of EETs and increase EET-mediated cardioprotective effects have demonstrated similar benefits in models of cardiac hypertrophy and HF.^176,177^ Animal models investigating EET-mediated cardioprotection in models of NICM are becoming more common. However, as with many of the CYP-derived eicosanoids, clinical data remains scarce, and truly translational studies are required to determine whether the CYP-sEH pathway is a safe and manipulatable target for human therapy.

##### CYP-derived EETs and diabetic cardiomyopathy (DCM)

Metabolic syndrome and diabetes begin an inflammatory cascade that is crucial to the development of cardiomyopathy. Individuals with either type 1 or type 2 diabetes mellitus (T1DM or T2DM) are at greater risk for cardiovascular complications and resultant mortality in non-diabetic subjects.^178,179^ While diabetes alone carries a risk for heart disease, T2DM is often coupled with other comorbidities such as obesity and metabolic syndrome that additionally complicate the prevention, treatment, and prognosis of patients that go on to develop DCM.^178^ DCM describes diabetes-related changes in the heart that are separate from CAD and hypertension associated forms of CVD. In diabetes and DCM, inflammation plays a key role and leads altered endothelial cell function, which in turn promotes vascular remodeling, resulting in atherosclerosis and ischemia. Eventually, inflammation induces cardiomyocyte apoptosis, leading to more profound cardiomyopathic changes. At the cellular level, studies have shown that the myocardium suffers from altered substrate utilization, lipotoxicity, increased oxidative stress, and interstitial fibrosis. Lipotoxicity basically describes the storage of fat in organs other than adipose tissue and plays a key role in these events and is also a contributing factor to the development of insulin resistance. Diabetic hearts have decreased myocardial GLUT4, glycolysis, and glucose oxidation, while there is a coincident increase in pyruvate dehydrogenase kinase, β-oxidation, and myocardial oxygen consumption, all of which reflects an increase in fatty acid metabolism secondary to decreased glucose utilization.^180^ In db/db and ob/ob mouse models of T2DM, the myocardium undergoes changes in mitochondrial morphology and develops mitochondrial uncoupling, leading to reduced ATP synthesis.

As lipid mediators involved in inflammation, hypertension, and glucose homeostasis, EETs are a viable method to protect against DCM. Also, in this situation, the cardiac-specific overexpression of CYP2J2 has beneficial effects on DCM and insulin resistance in high-fat diet-fed, low-dose streptozotocin-treated mice.^181^ In particular, the overexpression of CYP2J2 resulted in the maintenance of contractile activity, improved heart-specific glucose uptake, and insulin sensitivity, and attenuated the hypertrophy associated with diabetes. Also in this case, the molecular mechanisms underlying these effects were related to insulin-like growth factor 1 (IGF-1), insulin receptor substrate-1 (IRS-1), PI3K, AKT, AMPK, and PPARγ. CYP2J2 over-expression also attenuated increased PDK4 expression, which has been suggested to contribute to DCM by decreasing the pyruvate dehydrogenase complex.^181^

Ultimately, these studies suggest EETs retain their cardioprotective effects in DCM and may be a useful therapy for patients diagnosed with co-morbidities of diabetes and CVD. Finally, further research in this area is needed to determine whether EETs can be utilized in humans as a cardioprotective strategy against DCM.

#### CYP-derived EETs and vascular function

Local vascular tone is determined by a variety of factors such as neurotransmitters released from autonomic nerves, circulating vasoactive compounds, tissue metabolites, and endothelium-derived autacoids. The best-characterized vasodilator autacoids are nitric oxide (NO) and prostacyclin (PGI_2_), but a substantial component of the vasodilator response observed in response to receptor-dependent agonists or increases in flow is insensitive to inhibitors of NO synthases and COXs. Since the NO/PGI_2_-independent vasodilatation originally described was co-incident with vascular smooth muscle hyperpolarization, and was abolished by depolarizing concentrations of potassium, it was proposed to be mediated by an “EDHF”.^182^ Campbell et al.^19^ first reported that EETs relax precontracted coronary artery smooth muscle cells, induce cell hyperpolarization by increasing the open-state probability of Ca^2+^-activated K^+^ channels, and identified EETs as an EDHF. Shortly thereafter, the downregulation of a CYP2C enzyme in porcine coronary arteries was demonstrated to abrogate, NO, and PGI_2_-independent relaxation and hyperpolarization.^183^ Subsequent studies have demonstrated that the hyperpolarizing effects also exist in peripheral arteries,^184^ which indicated that CYP-derived EETs elicit vasodilation and improve vascular function in many stress conditions.

##### CYP-derived EETs and blood pressure

Hypertension is the most prevalent CVD and afflicts one in every three adults worldwide.^185^ Several factors contribute to chronic blood pressure elevation, which increases the risk for cardiovascular morbidity and mortality. Contributing factors to hypertension include elevated activity of the renin-angiotensin system, increased sympathetic activity, and inflammation.^185^ These factors result in excessive vasoconstriction and increased total peripheral resistance or impaired sodium excretion, increased extracellular fluid volume, and increased cardiac output.^22^ In many types of hypertension, EET levels are reported to decrease, an effect attributed to an increase in vascular sEH expression.^177^

The contribution of CYP eicosanoids to high blood pressure and the associated risk factors has been evaluated in hypertensive animal models as well as in humans. Overexpression of CYP enzymes attenuates the development of hypertension and improves cardiac function in spontaneously hypertensive rats, partly by EGF receptor (EGFR)-dependent effects on ANP.^163^ Human studies provide evidence that decreased EET levels result in elevated blood pressure,^186^ as CYP2C gene variants generate fewer EETs and are positively correlated with an increased risk for essential hypertension.^187^ Consistent with all these findings, increasing EET levels in animal models of hypertension decreases blood pressure and exerts cardiovascular protective actions.^177^ It therefore seems safe to say that decreased EET production (especially when associated with increased AngII) appears to be a contributing factor to hypertension.^177,188,189^

It is not just altered vascular production that contributes to hypertension, as CYP enzymes and the sEH are also expressed in the kidney and affect naturists. There is extensive evidence for an important contribution for EETs in maintaining kidney vascular and epithelial function.^18,190,191^ For example, EETs act to dilate preglomerular afferent arterioles and inhibit epithelial sodium channels (ENaC).^192^ A decrease in EET levels leads to excessive afferent arteriolar constriction and enhanced ENaC activity and salt absorption, which increases blood volume and blood pressure.^193^ Indeed, 11,12-EET can inhibit cortical collecting duct ENaC and increase sodium excretion. Conversely, EETs can lower blood pressure by inhibiting sodium absorption in the proximal tubule and cortical collecting duct.^194^ Importantly, excessive afferent arteriolar constrictor reactivity in hypertension is eliminated by sEH inhibition to increase kidney EET levels.^191^ Some models of hypertension can even be linked to changes in specific CYP enzymes asn salt-sensitive hypertension occurs when the kidney and vascular expression of CYP2C23 and CYP2C11 fail to increase in response to a high salt diet.^191^ In accordance with these findings, the genetic deletion of CYP2C23 (CYP2C44) in mice results in decreased kidney and vascular EET levels and salt-sensitive hypertension.

##### CYP-derived EETs, atherosclerosis, and coronary artery disease

Polymorphisms in the CYP2J2 gene have been shown to affect CAD risk and incidence in specific populations.^195,196^ One of the most relevant polymorphisms in terms of frequency and functional importance is located at −50 (G-50T) in the proximal promoter of CYP2J2. Screening of 289 patients with coronary artery disease and 255 control subjects revealed 77 individuals with the G-50T SNP (17.3% of CAD patients, 10.6% of control subjects; *P* = 0.026). The association of the G-50T polymorphism remained significant after adjustment for age, gender, and conventional cardiovascular risk factors (OR, 2.23; 95% CI, 1.04–4.79). The G-50T mutation resulted in the loss of binding of the Sp1 transcription factor to the CYP2J2 promoter and resulted in a 48.1 ± 2.4% decrease in CYP2J2 promoter activity (*P* < 0.01). Plasma concentrations of stable EET metabolites were significantly lower in individuals with the G-50T SNP.^195^ In addition, the presence of the CYP2J2*7 allele in an African-American population was associated with a significantly lower risk of incident CAD, while an increased risk of CAD along with lower plasma EET levels were observed in a Caucasian population^195^ Interestingly, EPHX2 polymorphisms have been linked to risk for coronary artery calcification and disease in young adults.^197^

In atherosclerosis-prone apolipoprotein E (ApoE)-deficient mice, recombinant adeno-associated virus (rAAV)-mediated CYP2J2 gene overexpression, which is associated with increased EET levels, prevented the development of high-fat diet-induced atherosclerosis.^198^ Mouse models of atherosclerosis have been relatively extensively studied and treating ApoE^−/−^ mice with sEH inhibitors prevents atherosclerosis induced by a high cholesterol diet.^199^ Similarly, studies in sEH^−/−^ mice have demonstrated a contribution for EETs to oppose vascular inflammation, atherosclerosis, and vascular remodeling.^177^ Moreover, sEH^-/-^ mice and animals with endothelial cell-specific overexpression of CYP2C8 or CYP2J2 demonstrate decreased vascular inflammation and NF-κB activity when exposes to endotoxin.^18^ EET-positive actions to attenuate atherosclerosis has been associated with decreased adhesion molecules and inflammatory cytokines.^18^ Thus, EETs and sEH inhibition decrease inflammation and have vascular protective actions that can combat atherosclerosis. The effects extend to abdominal aortic aneurysms.^200^ In particular, CYP2J2 overexpression could be linked with attenuated matrix metalloproteinase expression and activity, elastin degradation, and AAA formation, which was associated with reduced aortic inflammation and macrophage infiltration. Again, these effects were linked with the activation of PPARγ,^200^ but the same mice also manifested lower low-density lipoprotein and elevated high-density lipoprotein cholesterol levels, as well as attenuated expression of pro-inflammatory genes and proteins.^201^ These effects were associated with a reduction of serum lipid, interleukin (IL)-6, murine IL-8-KC, and IL-1α, and downregulation of gene expressions of ICAM-1, VCAM-1, and IL-6 in the arterial wall.^200,202,203^

Collectively, the beneficial effects of EETs and sEH inhibitors in the preclinical model were vasodilation, anti-hypertension, anti-inflammation, improved endothelial function, and lipid regulation. Moreover, the clinical association of sEH gene polymorphisms towards increased risks of atherosclerotic vascular disease provides a strong rationale to target sEH in the treatment of atherosclerosis.^204^

##### CYP-derived EETs and stroke

EETs or sEH inhibition protects either the heart or brain from the damage that occurs following an ischemic event.^21,152,156,205^ This protective action for EETs appears to be multifactorial and EETs likely inhibit apoptosis in the brain tissue. Brain tissue EET cell signaling antiapoptotic mechanisms involve increased Bcl2, ceramide inhibition, and decreased ROS.^156,206^ Indeed, we found that CYP2J2 overexpression increased EET productions, increases regional cerebral blood flow (rCBF) and microvascular density, decreased ROS production, decreased brain infarct size and apoptosis after ischemia, the effects of which were associated with increased activation of the PI3K/AKT and apoptosis-related protein in the ischemic brain. Liu et al.^207^ found that exogenous administration of 14,15-EET or AUDA could suppress astrogliosis and glial scar formation, inhibit microglia activation and inflammatory response, promote angiogenesis, attenuate neuronal apoptosis and infarct volume, and further promote the behavioral function recovery after focal ischemia.

Moreover, sEH was widely expressed in spinal cord tissue, mainly confined to astrocytes, and neurons. Administration of sEH inhibitor AUDA significantly suppressed local inflammatory responses as indicated by the reduced microglia activation and IL-1β expression, as well as the decreased infiltration of neutrophils and T lymphocytes.^208^ Furthermore, treatment of AUDA improved angiogenesis, inhibited neuron cell apoptosis, alleviated demyelination and formation of the cavity and improved motor recovery.^208^ In addition, epidemiological data demonstrating genetic polymorphism in the EPHX2 are associated with increased risk for ischemic stroke.^197^ We firstly found that there was a significant interaction between the EPHX2 G860A polymorphism, smoking and ischemic stroke risk such that nonsmokers carrying the EPHX2 G860A variant allele were at the lowest risk of ischemic stroke.^209^

These results together suggest that epoxyeicosanoid signaling and she inhibition are promising multi-mechanism therapeutic targets for the treatment of stroke.

##### CYP-derived EETs and angiogenesis

Angiogenesis is a complex process that involves the proliferation, invasion, and migration of endothelial cells to form tubes or primitive capillaries. Epoxides of AA have a clear link to angiogenesis.^74,210,211^ Munzenmaier et al.^212^ firstly found the link of CYP-EETs/sEH axis and angiogenesis, in which EETs promoted proliferation and tube formation in cerebral capillary endothelial cells released by cultured astrocytes. This fit well with observations that the overexpression of CYP2C9 and the corresponding production of EETs promoted the activation of the mitogen-activated protein 1 (MKP-1) mediated dephosphorylation and inactivation of c-Jun N-terminal kinase (JNK), effects ultimately culminating in the expression of cyclin D1 and proliferation in human endothelial cells.^213^ In addition, 11,12-EET-induced transactivation of the EGF receptor and activation of Akt kinase were inhibited by sphingosine kinase (SK) specific inhibitor.^214^ Activation of AKT by EETs was also linked to PI3K, inhibition of the forkhead factors FOXO1 and FOXO3a and subsequently a decrease in the expression of the cyclin-dependent kinase inhibitor p27kip1. The transfection of CYP2C9 overexpressing cells with either a dominant-negative AKT or a constitutively active FOXO3a inhibited CYP2C9-induced endothelial cell proliferation.^215^ In addition to the PI3K/AKT pathway, the inhibition of MAPKs was also found to attenuate EETs-induced endothelial proliferation.^74^ Work from Capdevila’s team further underscored that activation of p38 MAPK is required for the proliferative responses to 8,9- and 11,12-EET, and activation of PI3K is necessary for the cell proliferation induced by 5,6- and 14,15-EET.^216^ Moreover, treatment with EETs and the sEH inhibitor trans-4-[4-(3-adamantan-1-ylureido)cyclohexyloxy]benzoic acid^50,51^ (t-AUCB), respectively, significantly increase VEGF production,^217^ an effect prevented by CYP inhibitors.^218^ That is, multiple signaling pathways are involved in pro-proliferation effects of CYP-EETs/sEH system on endothelial cells.

Meanwhile, it is important to note that angiogenesis can be stimulated when EETs are generated by endothelial cells themselves, as well as when they were applied exogenously or generated from astrocytes. This indicates that the actions of the EETs cannot be restricted to an autocrine role but that a sufficient EET concentration must be able to leave the cell of origin to elicit paracrine actions on other cells. The development of novel transgenic animals has helped to confirm the effects of CYP-derived metabolites of AA on angiogenesis and vascular repair, e.g., in an ischemic rat hind limb model in which the overexpression of different CYP enzymes, including CYP2C11 and 2J2, was found to increase muscle capillary density.^74^ However, it remains unclear whether these pathways are linked to each other or are simply activated in parallel.

Endothelial cell migration is an essential step to form vessel-like structures.^219^ EETs promote endothelial cell migration by a mechanism thought to involve the endothelial NO synthase, MAPK, and the PI3K activation.^74,220^ The situation appears to be somewhat different in murine pulmonary endothelial cells in which 5,6- and 8,9-EET (but not 11,12- or 14,15-EET) evoke a MEK/MAPK and PI3K-dependent cell migration.^216^ Prior to migration out of a preexisting mature vessel, endothelial cells need to degrade the surrounding extracellular matrix and inhibit migration and proliferation of vascular smooth muscle cell,^221^ thus in turn providing space for the migration of endothelial cells and the diffusion of key growth factors, such as FGF-2, PDGF, and VEGF.^222,223^ A series of enzymes including collagenases, gelatinases, stromolysins, metalloelastases, and membrane-type matrix metalloproteases (MT-MMP), are responsible for the degradation of the extracellular matrix.^222^ Both 11,12- and 14,15-EET have been reported to activate one or more metalloproteases^220,224^ and promote the release of heparin-binding EGF-like growth factor (HB-EGF) from the cell surface.^225,226^ In addition, the sEH inhibitor (12-(3-adamantan-1-yl-ureido)-dodecanoic acid or AUDA) also reduced the protein expression of MMP-9 in ECs^227^ and MMP activity was increased in CYP-2C9-overexpressing cells increased and correlated with invasion ability.^220^

The formation of cord-like structures and primitive tubular structures are more direct evidence for angiogenesis. The overexpression of CYP2C9 in and/or the application of 11,12- or 14,15-EET to monocultures of endothelial cells have been linked to the formation of such structures in vitro on matrigel or in fibrin gels.^226,228^ The in vivo data also rapidly supported these and EETs-induced angiogenesis in the chick chorioallantoic membrane,^226^ as well as in EET-impregnated matrigel plugs in adult rats^228^ and in an ischemic rat hind limb model. In these models above, the overexpression of different CYPs, including CYP 2C11 and 2J2, was found to increase muscle capillary density.^74^ The potential mechanisms of EET-induced angiogenesis include that inhibition of the forkhead transcription factor to downregulate p27Kip1,^215^ crosstalk to EGF receptor,^226^ induction of FGF2^73^ and VEGF,^229^ often demonstrated via AKT activation,^215,226^ SRC-activation of STAT3,^229^ the activation of sphingosine kinase-1,^214^ and the induction of endothelial nitric oxide synthase.^74,219^ Moreover, EET-induced angiogenesis also involves crosstalk with other AA metabolizing pathways as 11,12-EET induced the expression of COX-2 in endothelial cells via a PKA-cAMP-dependent pathway^230^ and COX-2 protein shifted EET metabolism away from DHETs and towards epoxy hydroxyeicosatrienoic acids (EHETs) which have been attributed angiogenic properties.^231^ Which of these pathways is applicable probably depends on the species, type of endothelium, and EET regioisomers produced by the CYP epoxygenase.^232^

Other non-negligible events in the process of angiogenesis are an adaptation to hypoxia and the differentiation of endothelial precursor cells. This is particularly relevant in the tumor microenvironment (TME) when the pO2 drops once a tumor grows beyond a size where O2 needs can be met by discussion from outside the tumor. Hypoxia stimulates the expression of a series of CYP enzymes in endothelial cells including CYP2C8 and CYP2C9 to increase EET formation.^220,233^ Importantly, the same stimulus suppresses the expression of the sEH, at least in the mouse liver and a human hepatoma cell line^234^ to further increase EET levels. Consistently, hypoxia-induced angiogenesis in vitro was abolished by antisense oligonucleotides directed against CYP2C enzymes as well as by the CYP inhibitor MS-PPOH and the EET antagonist 14,15-epoxyeicosa-5(Z)-enoic acid (EEZE)^220,233^ and enhanced by the endothelial cell-specific overexpression of CYP2J2 or by sEH inhibitors around the ischaemic area in MI model.^142,235^ Exogenous EETs may even improve diabetic/non-diabetic wound healing caused by ischemia via modulating inflammation and angiogenesis.^224,236^ Endothelial precursor cells arising from hematopoietic stem cells in the bone marrow; upon proangiogenic stimuli, they proliferate, migrate, and differentiate into mature endothelium in several diseases such as myocardial ischemia, stroke, and in tumor growth and progression.^237^ Increasing EETs levels with t-AUCB promoted EPCs activation in the AMI patients via a PPARγ dependent manner.^238^ In addition, aerobic exercise modulated circulating EPC function via elevating EET concentrations in mice with AMI^239^ Thus, CYP-derived EETs promote angiogenesis via various mechanisms.

#### CYP-derived HETEs in CVD

CYP enzyme-dependent ω-hydroxylation of AA is a prototypic metabolic reaction of CYP4 family members that is important for hydroxyeicosatetraenoic acid generation. 20-hydroxyeicosatetraenoic acid (20-HETE) is the main product of the reaction catalyzed by three main CYP4 enzymes, i.e., CYP4A11, CYP4F2, and CYP4F3B. Multiple researches have linked 20-HETE with cardiovascular disorders and renal system. 20-HETE has been suggested to mediate androgen-induced hypertension through increasing the level of Cyp4a12^240^ and the overexpression of the Cyp4a12-20-HETE synthase in proximal tubular promotes salt-sensitive hypertension in male mice.^241^ In the kidney, however, 20-HETE exerts anti-hypertensive effects through inhibition of sodium reabsorption in the proximal tubule and thick ascending limb of Henle.^242^ Furthermore, 20-HETE acts as a vasoconstrictor of vascular smooth muscle cells by promoting calcium entry into cells to enhance phosphorylation of contractile elements.^243,244^ Several studies have suggested an interplay between 20-HETE and the renin–angiotensin aldosterone system (RAAS) in hypertension. Briefly, angiotensinogen II has been reported to increase renal production of 20-HETE, and 20-HETE can activate the RAAS by inducing angiotensin-converting enzyme.^245–247^ CYP4A was also reportedly upregulated in models of doxorubicin-induced cardiotoxicity with a consequent increase of 20-HETE synthesis.^248^ Furthermore, Jarrar et al.^249^ found that heart cyp4a12 was highly upregulated in mice after cardiac toxicity induced by NSAIDs. Thus, targeting of 20-HETE synthesis through manipulation of CYP4 enzymes could be considered in the future development of the drug for CVDs.

#### EET receptors

A mount of data has contributed to the characterization and understanding the role of CYP-derived metabolites function within CVD. However, the identity of the specific receptor(s) involved in epoxylipid responses remains unclear. Given that high-affinity EET binding sites on the surface of some cells, such as monocytes, vascular smooth muscle cells, and endothelial cells, many investigators have speculated that a specific EET receptor may exist on the membrane of cells.^182^ For instance, the 11(R),12(S)-EET is a more potent activator of renal artery KCa channels^250^ than 11(S),12(R)-EET. Also, in endothelial cells 11(R),12(S)-EET could induce the membrane translocation of TRPC channels rapidly while the other EETs (such as 14,15-EET and 11(S),12(R)-EET) were ineffective.^182^ In addition, many evidences suggest the actions of EETs are in part mediated via G-protein-coupled receptor (GPCR) signaling. For instance, biochemical studies have already indicated the importance of Gs Proteins in 11,12-EET-initiated signaling,^251^ and in endothelial cells the downregulation of Gs but not Gq/11 was recently shown to abrogate the effects of 11(R),12(S)-EET on TRPC6 channels.^252^ In addition, in HEK293 cells, G protein-coupled receptor 40 (GPR40) was also reported to be involved in mitogenic responses to EETs.^253^ GPR40 is an interesting candidate EET receptor, in which the medium and long-chain fatty acids are thought to be putative ligands. However, it remains inconclusive whether EET-induced changes in cAMP signaling as a response to classical GPCR cellular responses.^252^ In addition, it has been reported EETs can induce vasodilation via antagonizing thromboxane (TP) receptors in the vascular system.

Numerous reports illustrate the effects of PPARα and PPARγ activation with EETs. PPARs are involved in regulating lipid metabolism, inflammation, immune function, cell proliferation, and insulin secretion.^139,182^ Therefore, it is more than likely that these intracellular lipid mediators interact with intracellular receptor molecules such as the PPAR family of nuclear receptors. The significance of PPAR activation in mediating effects of EETs needs further investigation to draw a clear mechanistic pathway.

## AA metabolisms in cancer

Cancer is a major health burden worldwide and represents one of the leading causes of mortality and morbidity, with ~14.1 million new cases and 8.2 million cancer-related deaths annually.^254^ Despite the advance in various treatment strategies, such as combinations of surgical resection, radiation or chemotherapies and immune therapies, the 5-year survival rate for some cancers is still relatively low. Furthermore, the underlying cause(s) of cancer remain unclear. Thus, there is an unmet need to develop an effective strategy for preventing the development of this devastating disease. While the results of large chemoprevention trials thus far have not been encouraging, a 20-year follow-up study with aspirin, a non-steroidal anti-inflammatory agent that acetylates and inhibits COX-2, showed that the mortality rates from all solid cancers were 20% lower for those receiving aspirin, with adenocarcinoma being the most reduced (34%).^255,256^ This is strong evidence for the role of anti-inflammatory agents such as COX inhibitors in cancer prevention. Probably the COX metabolites with the highest tumorigenic and metastatic potential is PGE_2_, as it inhibits cancer cell apoptosis and increases invasiveness as well as promoting angiogenesis^257^ in tumors. The pathways implicated include mTORC1/VEGR, NF-κB, MAPK/JNK/p38, PI3K/Akt as well as epigenetic modifications.^258–260^ There also seems to be a role for CYP-derived EETs in the development of various cancers.^261,262^

### Roles of COXs and their metabolites in cancer

Chronic inflammation is clearly associated with an increase in the risk of cancer.^263^ One of the strongest associations between chronic inflammation and cancer is the increased risk in individuals with inflammatory bowel diseases. Inflammation also appears to have an important role in the development of other cancers, for example, prostate, bladder, and pancreatic cancers. Chronic inflammation causes the upregulation of a number of inflammatory cytokines including IL-1β, IFNγ, and TNFα. The NF-κB pathway is activated in many chronic inflammatory states, and evidence directly links the NF-κB pathway to increased tumor formation and inflammation in experimental mouse models of intestinal cancer.^264–266^ Because NF-κB plays a role in COX-2 regulation at the transcriptional level, prostaglandin H synthase or COX-2 expression is increased, and higher levels of inflammatory PGs are formed.^267^ Diminished expression of 15-prostaglandin dehydrogenase (15-PGDH), a prostaglandin degradation enzyme also contributes to the elevated PG levels in cancer.^266,268^ The aberrant AA metabolism observed in cancer cells results in a high concentration of PGs, in particular, PGE_2_.^41,269^ Because of the high concentrations of PGE2 in tumors, many investigations have focused on the EP receptors.^266,270^ Indeed, EP2 expression is upregulated compared with normal tissues in colorectal and breast cancers.^116,266,271^ Moreover, both EP2 and EP4 mRNA was upregulated in human glioblastomaastrocytoma U373 MG cells compared to the primary astrocytes.^272^ The deletion of the EP2 receptor in APC/Min mice substantially reduced polyp formation,^271^ while deletion of the EP4 receptor has been shown to decrease the formation of aberrant crypt foci in animals treated with the colon carcinogen azoxymethane.^273^ At the level of signaling, the EP2/4 receptors are G protein-coupled receptors and PGE2 can thus activate PKA to stimulate several divergent pathways to mediate pro-tumorigenic activities.^274^ For example, PKA phosphorylates GSK-3, to alter the APC/β-catenin/TCF pathway, which regulates cell proliferation, angiogenesis, and apoptosis.^256,274,275^ PGE2 also can transactivate the EGF receptor, increase amphiregulin, and enhance the RAS-MAP kinase pathway, and transactivate the PPAR δ pathway.^276–279^

Numerous epidemiological, clinical, laboratory, and animal and cell culture studies confirm that the use of COX inhibitors or nonsteroidal NSAIDs is effective at inhibiting the incidence and mortality of colorectal cancer.^280,281^ In addition to colorectal cancer, NSAIDs have also been associated with a reduced risk of breast, esophageal, stomach, bladder, ovary, and lung cancers.^282–284^ Despite the extensive studies on the effectiveness of NSAIDs as chemopreventative agents, the molecular mechanisms underlying their chemopreventative effects are not well understood. While is was initially presumed that the anti-cancer activity of the NSAIDs could be attributed to the inhibition of COX-1/COX-2, this concept has been challenged by the fact that very high doses of COX inhibitors are frequently required to exhibit tumor inhibitory effects but only low doses are required to prevent PG generation.^266,285^ Therefore, COX-independent effects may contribute to the chemopreventative activity of NSAIDs.^285^ There is at least circumstantial evidence for such an effect as NSAIDs inhibit the growth of colon cancer cell lines that do not express COX-1 or COX-2^286^ and inhibit the growth of mouse embryo fibroblasts lacking both the COX-1 and COX-2 genes.^287^

### Roles of LOXs and their metabolites in cancer

The inhibition of COX activity by NSAIDs makes the substrate, i.e., AA, available for metabolism by other enzymes and may cause a shift in the AA metabolite profile from PGs to LOX-derived hydroxylated lipids. 5-LOX, 12-LOX, 15-LOX-1, and 15-LOX-2 are reported to have some influence on tumor development. For example, there are numerous reports of increased 5-LOX expression in cancer cells, e.g., 5-LOX and 5-LOX activating protein (FLAP) was universally expressed in epithelial cancer cell lines,^288^ and 5-LOX was elevated in human pancreatic cancer cells^289^ as well as in malignant tissue from patients with prostate carcinoma. The latter study reported 2.2-fold greater levels of 5-HETE in malignant tumor tissue compared with benign tissue.^290^ Fitting with this. MK591, a specific 5-LOX inhibitor-induced apoptosis in prostate cancer cells via downregulation of PKCε, a pro-survival serine/threonine kinase.^291^ Similarly, both 5-LOX mRNA and protein were higher in gastric cancer than non-tumor tissues and 5-LOX inhibition induced apoptosis in the human gastric cancer AGS cell line.^292^ Added to all this, the combined use of the 5-LOX inhibitor zileuton and the COX-2 inhibitor celecoxib elicited synergistic effects in human oral cancer and colon cancer suggesting that COX-2/5-LOX inhibitor may be a more effective direction of antitumor drug discovery.^293,294^ Indeed, licofelone, a potent COX-2/5-LOX inhibitor was shown to induce apoptosis in both androgen-dependent and androgen-independent prostate and colon cancer cells.^295,296^

15-LOX-1 is present in human colorectal cancer cells^216^ and converts AA to 15-HETE and linoleic acid to 13-hydroxyoctadecadienoic acid (13-HODE). Interestingly, 15-LOX-1 has been associated with anti-tumorigenic activity in human colorectal cells,^297^ and in human colorectal cancer.^298^ It is perhaps not surprising therefore that the expression of 15-LOX-1 is lower in human colorectal tumors than in normal tissue, and as a consequence, so are the levels of the major 15-LOX-1 metabolite, 13-HODE.^266,299^ How 13-HODE its anti-tumor effect is likely related to its ability to downregulate PPARδ,^300^ and stimulate the phosphorylation of the tumor suppressor gene p53, which results in increased expression of many downstream targets.^301^ However, while the growth inhibitory effects of 15-LOX-1 were p53 dependent, 15-LOX-1 metabolites failed to induce its phosphorylation and a 15-LOX-1 inhibitor did fail to prevent p53 phosphorylation.^301^ Such findings may indicate that an additional protein may be involved—the interaction of the 15-LOX-1 protein with the DNA-PK kinase which can phosphorylate p53^302^ could account for such a phenomenon.

12-LOX is the LOX isoform expressed in epithelial cells and myeloid cells including platelets. Many mutations in this isoform are found in epithelial cancers, suggesting a potential link between 12-LOX and tumorigenesis.^303^ Thus, the LOX, especially 15-LOX-1, appears also to have a role in the reduction of tumors by COX inhibitors.

Recently, Haribabu et al. showed reduced CD8^+^ T cell migration and increased tumor growth in BLT1^−/−^ mice injected with B16 melanomas, indicating the important role of BLT1 signaling in immune surveillance and anti-tumor immunity.^304,305^ In the murine spontaneous colon cancer model (ApcMin mice), the same authors also reported that BLT1^−/−^ Apc^Min/+^ mice showed increased intestinal tumor development, exacerbation of colon inflammation, and increased mortality.^304,306^ Furthermore, in acrystalline silica-induced lung cancer model, LTB4 production by inflammatory leukocytes increased macrophage phagocytosis and led to sustained activation of neutrophils via an autocrine loop of LTB4 production. Although LTB4-BLT1 signaling was shown to play a key role in anti-tumor responses, critically, the cell-specific roles of BLT1 in vivo are still unknown, and further studies that employ conditional cell-specific knockout of BLT1 are needed in these cancer models.

In addition, LTC4 and its metabolites LTD4 and LTE4 (together referred to as cysteinyl LTs, CysLTs) are inflammatory mediators derived from AA via the 5-LOX pathway.^1^ They exert many of their functions through the CysLT1 receptor, which is expressed in pulmonary smooth muscle and interstitial macrophages. CysLTs contribute to cancer progression and several observations support a pro-tumorigenic effect of LTD4 via CysLT1 in colorectal cancer.^307^ Montelukast is a CysLT1 receptor antagonist already used in asthma treatment.^308^ Interestingly, asthma patients treated with montelukast have a considerably lower risk to develop cancer.^309^ In animal studies, montelukast increased survival rates in a spontaneous metastasis model of Lewis lung carcinoma (LLC) and delayed tumor growth.^308,310^

### Roles of CYP dependent monooxygenases and their metabolites/sEH in cancer

Emerging evidence has demonstrated that CYP-derived EETs regulates multiple cellular processes of carcinogenesis and progression, including cell proliferation, survival, angiogenesis, invasion, and metastasis.^23,311,312^ CYP enzymes, such as CYP2J2 are highly expressed in various human carcinoma cell lines (including LS-174, ScaBER, SiHa, U251, A549, Tca-8113, Ncl-H446, and HepG2) and human tumors (including esophageal adenocarcinoma, pulmonary carcinoma, breast carcinoma, stomach carcinoma, liver carcinoma, and colon adenocarcinoma). In animal models CYP2J2 overexpression promoted cancer growth and metastasis,^261^ and CYP enzyme-derived EETs enhance tumor cell motility, invasion, adhesion and metastasis.^262^ These studies were a prelude to a wave of subsequent studies reporting the relationship of the CYP-EET/sEH axis and cancer development.

#### Levels of CYP-EETs in cancer

The high expression level of CYP enzymes implies that AA epoxide levels should also be increased in tumors. EETs were first detected in colonic adenocarcinoma homogenate back in 1995.^313^ However, because of the chemical instability the measures of the level were much lower than the actual values. This problem led some researches to use DHET levels as an indirect index of EET content, an assumption that certainly held true for cancer cell lines versus HEK-293 cells.^261^ Also, DHET levels were significantly higher in urine and plasma from patients with leukemia/lymphoma than from healthy volunteers.^314^ The elevated DHET could indirectly imply elevated sEH activity or expression and pretreatment with sEH inhibitors has been reported to significantly improved the stability of EETs in various types of biological samples.^315,316^ Since these early reports the development of methodology, especially ultra-high performance liquid chromatography-tandem mass spectrometry (UHPLCMS/MS) has made EET analyses more specific and sensitive.^317,318^ Using such techniques, EETs have been detected in tumor tissues or cells in various conditions. Conversely, inhibition of EETs generation is the key link to uncover novel approaches for tumor treatment.^319,320^

#### Polymorphisms of CYP epoxygenases in cancer

Genetic polymorphisms of CYP enzymes and the sEH, including single nucleotide polymorphisms, gene duplications, and deletions, resulting in abolished, reduced, altered, or increased expression and activity (Table 2). It is worth emphasizing that changes in AA-derived EETs and anti-tumor drugs due to CYP polymorphisms have been related to cancer susceptibility, tumor characteristics, and treatment response. Therefore, there is no doubt that CYP polymorphisms are closely associated with cancer fate.

**Table 2:** Gene polymorphism of several human CYP epoxygenases and tumor risk

CYP gene	Important variants	rs Number	Type of variation	Effect on enzyme activity	Cancer risk	Reference
CYP2J2	CYP2J2*7	rs890293	-50G > T(-76G > T)	Disrupted Sp1 site	–	^196,326^
CYP2C8	CYP2C8*2	rs11572103	805A > T (Ile269Phe)	Unchanged (AA) Activity reduced (Paclitaxel)	–	^333^
	CYP2C8*3	rs11572080 rs10509681	416G > A (Arg139Lys) 1196A > G (Lys399Arg)	Activity reduced Activity reduced	Early breast cancer-related events (higher); Colorectal cancer risk (no association).	^332–334^
CYP2C9	CYP2C9*2	rs1799853	430C > T (Arg144Cys)	Activity reduced	Non-small cell lung cancer (lower); Head and neck squamous cell carcinoma (higher); colorectal cancer (contradictory); bladder cancer (lower); breast cancer (higher)	^346,347^
	CYP2C9*3	rs1057910	1075A > C (Ile359Leu)	Activity reduced	Above	Above

**CYP2J2**: At least 9 variants of CYP2J2 have been identified, i.e., CYP2J2*2 to *10 (http://www. https://www.pharmvar.org). CYP2J2*2, *3, *4, and *6 carry A4274G, C4724T, T5754A, and A12104T mutations, leading to a statistically significant decrease in AA metabolism in vitro. These mutations result in 59%, 41%, 30%, and 5% of the wild-type CYP2J2 activity, respectively.^321,322^ CYP2J2*7 has a G4T substitution in the regulatory region at position-76 (50) of transcription start and lacks the binding site for Sp1, and consequently lowering CYP2J2 protein and its metabolites in vivo.^193,195^ CYP2J2*8 was reported in Koreans with frequencies of 0.8% in 2005,^323^ and has a point mutation in exon 6 (G9344A), resulting in the almost complete loss of enzyme activity.^323^ CYP2J2*10 carries a C3444A mutation in exon 2 was found in one fetal tissue with unknown ethnicity in 2006 and possibly severely damaged CYP2J2 protein activity.^251^ In contrast, there is no apparent difference between CYP2J2*5 and *9 which carry the G10244A and P3514L mutations, and wild-type CYP2J2—at least as far as AA metabolism is concerned.^322,323^ Most research on the relationship between CYP2J2 polymorphism and disease has focused on the cardiovascular system instead of neoplastic disease. For example, two intronic CYP2J2 SNPs (rs10889160 and rs11572325) were associated with an increased risk of MI.^324^ In addition, the most common variant (CYP2J2*7) with the frequency of 1.1–1.2% in a Russian, 2.6% in Chinese, and 11–17% in Africans increased the risk of hypertension and MI,^195,325^ is linked with a diminished capacity to synthesize EETs.^326^ Tumor development and cardiovascular benefits are often contradictory when assessing CYP2J2-EET functions. Thus, we speculate that the loss of function of CYP2J2 polymorphism, which detrimental to cardiovascular health, may decrease the risk of neoplastic disease.

**CYP2C8**: CYP2C8, which constitutes 7% of total hepatic microsomal,^327^ is responsible for the oxidative metabolism of at least 5% commonly used clinical drugs, including the anticancer drugs paclitaxel, cyclophosphamide and ifosphamide and Imatinib.^328–330^ Regardless of the frequency, 14 polymorphic variants in CYP2C8, referred to as CYP2C8.2 through CYP2C8.14 (http://www.cypalleles.ki.se/cyp2c8.htm) and an unclassified form named CYP2C8 P404A^331^ have been reported. Of these, CYP2C8*2 (805A > T, Ile269Phe) and CYP2C8*3 (416G > A/1196A > G, Arg139Lys/ Lys399Arg) are two major variant alleles with 4–18% frequency in Africans and 10–23% frequency in Caucasians, respectively.^332^ Both variants demonstrate decreased enzymatic activity for paclitaxel 6a-hydroxylation, leading to a corresponding increase in drug exposure in paclitaxel-treated patients.^330,333^ Patients carrying CYP2C8*3 are more likely to achieve complete clinical response to neoadjuvant paclitaxel treatment but have the risk of severe peripheral neurotoxicity.^334^ The N-demethylation of imatinib, the key drug for patients with chronic myeloid leukemia, is also mainly mediated by CYP2C8, for which the CYP2C8*2 and CYP2C8*3 have a gain-of-function effect on imatinib while CYP2C8*4 polymorphism was opposite.^335,336^ In addition to influencing the pharmacokinetics and pharmacodynamics of those anticancer drugs, the question of whether CYP2C8 polymorphism affects the occurrence and development of tumors is also very important. The CYP2C8 genotype (rs1058930), those who have the CG allele, have a 7.74 degree increased risk of breast cancer (CI = 95% 0.95–62.5) in women in Mazandaran province.^337^ Moreover, the CYP2C8/9 *3/*1/*2/*1 genotype seem to be at higher risk of breast cancer recurrence (in tumors larger than 20 mm), especially in women treated with tamoxifen.^338^ However, the most common functional genetic variant i.e. CYP2C8*3 does not show a major association with colorectal cancer risk.^339^ CYP2C8*3 also demonstrated an impaired metabolism of AA to 11,12- and 14,15-EET, which were 26-45% that of wild-type CYP2C8*1.^333^

**CYP2C9**: CYP2C9 accounts for about 20% of hepatic CYP content and metabolizes about 10% of therapeutically relevant drugs such as the anticoagulant warfarin, the anticonvulsant phenytoin, the antidiabetic drug tolbutamide, and numerous NSAIDs.^340,341^ CYP2C9 also is involved in the bioactivation of several carcinogens such as polycyclic aromatic hydrocarbons (PAHs) and heterocyclic aromatic amines,^342,343^ and the generation of endogenous active substances, especially EETs,^344^ leading to be associated with cancer risk. Over 30 CYP2C9 alleles have been detected [http://www.cypalleles.ki.se/cyp2c9.htm]. Among them, the CYP2C9*2 (R144C) and CYP2C9*3 (I359L) variants occur at a high frequency among Caucasians with frequencies of 0.08–0.14 and 0.04–0.16, respectively,^345–347^ both yielding enzymes with decreased activity.^347^ Other variant CYP2C9 alleles with relatively low frequencies have also been reported, although no association studies between them and human cancer risk have been performed. An increased frequency of the CYP2C9*2 allele in patients with lung cancer has been found and was linked with an increased risk of lung carcinogenesis in a North-American population.^348,349^ Concerning colorectal cancer, individuals carrying CYP2C9*2 or *3 alleles are at increased risk of developing colorectal cancer possibly for CYP2C9-mediated metabolic activation of PAHs and heterocyclic aromatic amines and diminishing the protective effects of NSAIDs.^350,351^ In addition, a higher prevalence of cases with variant genotypes of CYP2C9*2 or *3 were associated with an increased risk to develop head and neck squamous cell carcinoma (HNSCC).^352^ Conversely, other studies found that CYP2C9 polymorphism did not show any association with the risk of lung or colorectal cancer in a Spanish population,^353,354^ and even decreased the risk of bladder cancer in a single case-control study with 958 cases and 1029 controls.^355^ Except for their actions on the risk of various cancers, functional variants in the CYP2C9 also altered the clinical impacts of anticancer drugs. For example, CYP2C9*2 heterozygotes increased the risk of an insufficient response to breast cancer neoadjuvant chemotherapy 4.64-fold higher (OR = 4.64, *p* = 0.02) than in patients with the wild-type allele.^356^ In addition, CYP2C9*2 and CYP2C9*3 metabolize AA less efficiently than CYP2C9*1 and that they play a role in the progression of non-small cell lung cancer (NSCLC) via impaired EET biosynthesis.^357^ Together, CYP epoxygenases (especially CYP2J2, CYP2C8, and CYP2C9) and AA-derived EETs were widely distributed in various tumors and play important roles in the initiation and development of cancer. Moreover, CYP polymorphisms are also closely associated with cancer fate.

#### CYP-derived HETEs and cancer

20-HETE has long been implicated in the proliferation of tumor cells and endothelial cells, often invoking the participation of growth factors, such as VEGF, epidermal growth factor (EGF), fibroblast growth factor (FGF), or platelet-derived growth factor (PDGF).^358^ Modulation of the CYP4:20-HETE pathway has very pronounced effects on tumor size in animal models of the brain, kidney, and breast cancer. For example, following implantation into normal rat forebrain of U251 glioma cells with CYP4A1 overexpression, a 10-fold increase in tumor volume was observed compared with the nontransfected cells.^359^ Similarly, chronic treatment with HET0016, a potent and selective CYP4 inhibitor, increased survival time by 5 days in 9L gliosarcoma tumors, apparently through a combination of reduced mitosis and increased apoptosis.^360^ Injection of mice with an NSCLC-derived cell line (A549) transfected with CYP4A11 increased the tumor size and growth rate, both of which were reversed by HET0016 or WIT002.^361^ In addition, several groups, applying independent experimental approaches, established a role for 20-HETE in angiogenesis in the early 2000s. In one of the first of these studies, angiogenesis induced in skeletal muscle by chronic electrical stimulation was accompanied by a 2.5-fold increase in a 20-HETE formation that could be completely blocked by HET0016.^362^ More recently, CYP4Z1 overexpression in breast cancer cells has been linked to increased VEGF expression, angiogenesis, cell proliferation, and migration in vitro as well as increased tumor weight in xenograft models.^363^ Recently, Zeldin and coworkers found that endothelial cells from CYP4F2 transgenic mouse exhibited a twofold increase in levels of 20-HETE, increased growth and tube formation with upregulation of VEGF and the prooxidant enzyme NADPH oxidase subunits (gp91phox and p47phox).^364^ In addition, endothelial progenitor cells (EPCs) express relatively high levels of CYP4A11 and 20-HETE,^365^ and in return 20-HETE has been shown to promote EPC angiogenesis both in vitro and in vivo.^365,366^ Collectively, these studies provide strong support for the CYP4:20-HETE pathway as a potential drug target for combating tumor growth and metastasis.

### AA cascade and cancer-associated signaling pathways

Crosstalk between AA pathway associated enzymes and their metabolites regulate many pathophysiological processes in cellular systems and within the TME.^367,368^ The main biological functions of AA metabolites in cancer cells were concluded in Table 3.

**Table 3:** Biological activities of AA metabolites in cancer cells

AA metabolites	Signaling pathways	Biological process	Cancer	Reference
PGE2	Activation of mTORC1, leading to VEGF production	Cell proliferation and angiogenesis	Colon cancer	^257^
	Prostaglandin E2 enhances intestinal adenoma growth via activation of the Ras-MAPK cascade	Cell proliferation	Intestinal adenomas	^377^
	Activation of MAPK/Erk pathway signaling and epidermal growth factor receptor	Cell proliferation	NSCLC	^377^
	via inactivation of p300 signaling and VEGF pathway	Cell proliferation and angiogenesis	Breast cancer	^520^
	Activation of EP2/EP4 receptor and PKA/ERKs signaling	Cell migration and metastasis	Pancreatic cancer, breast cancer	^274^
TXA2	Activation of TP, leading to CDK1/cyclin B1 expression via p38/JNK pathway	Cell proliferation	Multiple myeloma, NSCLC	^521^
LTB4	ROS induction via BLT1/NOX4 pathway, leading to activation of EGFR/PI3K/ERK1/2/c-Myc pathway	Cell proliferation and inflammation	Hepatoblastoma, Lung cancer	^522^
	LTB4 stimulates growth of human pancreatic cancer cells via MAPK and PI-3 kinase pathways	Cell proliferation	Pancreatic cancer	^376^
	Activation of BLT2, leading to vimentin expression via ERK2 activation	EMT and invasion	Pancreatic cancer	^523^
	Crystalline Silica-Induced Leukotriene B4-dependent Inflammation Promotes Lung Tumor Growth	Cell proliferation and inflammation	Lung cancer	^307^
12-HETE	Activation of 12-HETER, leading to RHO/ROCK/MYPT/MLC2 pathway	Cell migration and invasion	Colorectal adenocarcinoma	^524^
	Activation of ERK/p38/JNK pathway	Cell proliferation	Pancreatic cancer	^525^
LXA4	Inhibition of NF-κB, leading to inhibition of p53 and cyclin D1	Anti-proliferation and anti-migration	Cervical carcinoma	^526^
	Suppression of ROS, leading to inhibition of ERK/MMP-9/MMP-2 pathway	Anti-invasion	Pancreatic cancer	^527^
11,12-EET	Activation of EGFR and PI3K/Akt pathway	Invasion and migration	Breast cancer	^262^
	Induction of VEGF expression	Cell proliferation and angiogenesis	Breast cancer	^388^
14,15-EET	Induction of integrin αvβ3 expression, leading to activation of FAK/PI3K/Akt pathway	EMT and chemoresistance	Breast cancer	^528^
	Activation of STAT3, leading to nuclear translocation of STAT3	Cell proliferation and metastasis	Breast cancer	^411,529^

#### AA-COXs, -LOXs and cancer

Several cytokines, including pro-inflammatory cytokines, induce the expression of cytolic PLA2, COX-2, and 5-LOX genes through activation of the IκB kinase (IKK)/IκB/NF-κB/AP-1/p300 pathway.^369–372^ TNFα induced cPLA2, COX-2, and 5-LOX expression were shown to be mediated by TNFR/p42/p44 MAPK/Elk-1/p300 and p38 MAPK- and JNK1/2-dependent AP-1/p300 pathways in human lung epithelial cells.^369,371^ IL-1β also elicits similar effects. Another cytokine, i.e., IL-8 activates cPLA2 via MAPK signaling pathway in PMNLs.^8,373^ Eicosanoids (PGs and LTs) stimulate the expressions of cPLA2, COX-2, and 5-LOX genes via activation of G-protein-coupled receptors (GPCR) mediated MAPK/NF-κB signaling pathways in cancer or pro-tumorigenic cells.^374–378^ Eicosanoids (PGs and LTs) can stimulate or promote tumor epithelial cell survival, proliferation, invasion, and metastasis and inhibit apoptosis by modulating multiple signaling pathways.^1^ Ultraviolet (UV) irradiation from solar exposure is a risk factor for carcinogenesis, which activates AA pathway via MAPK and NF-κB/AP-1-mediated signaling pathway. Yan et al.^379^ reported that UVB-induced LTB4 production and 5-LOX expression. COX-2 pharmacologic inhibition and COX-2 gene knockout prevent UVB-induced SKH-1 mouse skin tumorigenesis.^8,380,381^ Chen et al. found that UVB-induces the expression of cPLA2, which is mediated by oxidative stress. Black et al.^382^ reported that UVB-upregulated expression of COX-2 and other enzymes involved in PGs synthesis and TXA2, and 5-LOX and other enzymes involved in LTs synthesis, along with pro-inflammatory cytokines, namely IFNγ, IL-1β, TGF-β, and TNF-α in human corneal epithelial cells. The same investigators also found that inhibition of p38 MAPK blocked UVB-induced expression of COX-2, 15-LOX-2, and TNF-α, which demonstrate that UVB induces expression of COX-2, LOXs, and cytokines like TNF-α via MAPK signaling pathway.^382^ UVA upregulates the expression of COX-2 gene through MAPK/AP-1 mediated pathways.^383^ TPA/PMA, a tumor promoter, induces expression of COX-2 by activating MAPK/NF-κB/AP-1 mediated pathways whereas COX-2 inhibitors suppress.^8,384,385^ In addition, crystalline silica can promote lung tumor growth mediated by LTB4/BLT-1.^386^

#### Mechanisms of AA-CYP-EETs/sEH on cancer

In 2005, our laboratory explored the potential roles of CYP2J2 and its active products EETs on the neoplastic phenotype of carcinoma cells for the first time.^261^ Overexpression of CYP2J2 or addition of EETs to cultured carcinoma cell lines in vitro markedly accelerated proliferation, cell counts, cell cycle, and protected carcinoma cells from apoptosis induced by TNF-α. At the molecular levels, this involved the phosphorylation of EGFR and activation of PI3K/AKT and the MAPK signaling pathway. In contrast, either the downregulation of CYP2J2 transfection or the addition of epoxygenase inhibitors inhibited proliferation and accelerated TNF-α-induced apoptosis. In addition, carcinoma cells overexpressing CYP2J2 generated tumors at a faster rate and resulted in larger tumors than those generated from control carcinoma cells in vivo xenograft tumor model.^261^ Similarly, EETs promoted proliferation and increased the number of cells in the S/G2-M phase in a dose- and time-dependent manner in four tumor cell lines. The later effects were abolished by the inhibition of PI3K, MAPKK, MAPK, and PKC.^387^ Moreover, a specific inhibitor of CYP2J2 decreased EET production by ~60%, and inhibited the proliferation of human tumor cells at the same time as increasing tumor cell apoptosis via a caspase-3, Bcl-2 and Bax-dependent mechanism.^388^ Addition of exogenous EET or CYP2J2 overexpression also markedly accelerated proliferation and attenuated apoptosis in cultured human-derived malignant hematologic cell lines, which could be blocked by the pretreatment with the CYP2J2 inhibitor.^314^ Similar pro-proliferative and anti-apoptosic effects of the EETs were also observed in pheochromocytoma/paraganglioma tumors.^389^

CYP2J2 and CYP2C9 expression has also been correlated to high Ki-67 labeling indices in adenocarcinoma (EAC) and squamous cell carcinoma (ESCC). Selective inhibition of CYP2C9 decreased tumor cell proliferation and led to a G0/G1 phase cell-cycle arrest in vitro, which was abolished by the addition of 11,12-EET.^388,390^ Moreover, CYP3A4 is a highly active AA epoxygenase and synthesized AA epoxygenase products 8,9-, 11,12-, and 14,15-EET in the breast cancer lines.^391^ CYP3A4 silencing blocked the cell cycle at the G2/M checkpoint and induced apoptosis in the MCF7 line via inhibiting Stat3 (Tyr-705) phosphorylation, thereby inhibiting anchorage-dependent growth and survival. Knockdown of CYP3A5 and -2C8, both of which exhibit homology with CYP3A4, inhibited the proliferation of the MCF7, T47D, and MDA-MB-231 lines to varying degrees.^391^ Also, overexpression of CYP3A4 promoted the cell growth and cell cycle progression from the G1 to the S phase in a human hepatoma cell line, which was attenuated by a putative EET receptor antagonist, 14,15-EEZE and a PI3K inhibitor.^392^ These results suggest that CYP3A4 activity can accelerate tumor progression, which is independent of the activation of carcinogens and metabolism of anti-cancer drugs.

EETs and CYP2J2 transgenic mice attenuate doxorubicin-mediated cardiac damage by protecting mitochondria.^151,393^ More recently, 11,12-EET was reported to increase the expression of the antioxidant enzymes superoxide dismutase and catalase, and to attenuate mitochondrial transmembrane potential collapse and caspase activation in Tca-8113 cancer cells induced by the anti-leukemia drug arsenic trioxide.^394^ In addition, stably overexpressed CYP2J2 in a breast cancer cell line reduced the production of reactive oxygen species (ROS), thereby preventing cell death from anti-cancer agents such as paclitaxel, doxorubicin, sorafenib, and staurosporine.^395^ The expression and activity of aldehyde dehydrogenase 1A1 (ALDH1A1) were strongly upregulated in the CYP2J2 expressing cells and ALDH1A1 gene silencing restored their sensitivity to paclitaxel.^395^ CYP3A4 was found to be required for tumor formation in ER^+^/HER2^−^ breast cancer by suppressing autophagy, in part, by inhibiting AMPK activation. The effect was also associated with mitochondria, where CYP3A4 promoted the activity of the electron transport chain and increased oxidative phosphorylation.^396^ CYP3A4 knockdown or inhibition by biguanides activated AMPKα, promoted autophagy, and prevented mammary tumor formation.^397^ These results indicate that AA metabolizing CYP epoxygenases and EETs also are associated with mitochondrial function and oxidative stress of cancer cells, which may be another potential mechanism of their anti-apoptosic actions.

Primary tumor formation is a necessary requirement for metastasis, and it is estimated that ~1 × 10^6^ cells per gram of primary tumor escape into circulation per day. However, only a fraction of cells leaving the primary tumor to survive in circulation and even fewer cells colonize secondary sites.^398^ Jiang and his colleagues indicated that the overexpression of CYP2J2 or the exogenous application of EETs significantly induced tumor cell migration, invasion, adhesion to fibronectin, as well as colony-forming capacity.^262^ Consistently, CYP2J2 overexpression also enhanced metastatic potential in vivo and rAAV CYP2J2-infected human breast carcinoma cells developed 60% more lung metastases in athymic BALB/c mice.^262^ Selective inhibition of CYP2J2 prevented tumor cell adherence, invasion, and migration by decreasing the activation of the EGFR and PI3K/AKT pathways in vivo.^388^ Actin-myosin microfilament formation is closely associated with the invasion and migration of cancer cells. 11,12-EET was found to induced prostate carcinoma cell spreading and the formation of actin-myosin microfilaments possibly by the trans-activation of EGFR and PI3K/AKT pathways, which could account for the observed effects on cell invasion and migration.^399^ Blocking EET synthesis or activation using EET antagonists such as 14,15-EEZE, on the other hand, caused the cells to become more rounded and smaller.^399^ Together, these data suggest that CYP inhibition may represent a novel approach to prevent metastasis of human cancers. In addition, endothelium-derived EETs also contribute to tumor metastasis. Briefly, endothelial-specific expression of either CYP2C8 or CYP2J2 (Tie2-CYP2C8-Tr, Tie2-CYP2J2-Tr) accelerated the escape from tumor dormancy and extensive multi-organ metastasis.^400^

The TME is composed of several distinct cell types, including fibroblasts, pericytes, immune cells, adipocytes, endothelial cells, and a noncellular compartment, the extracellular matrix. The cross talk between cancer and stromal cells in the TME promotes does much to create optimal conditions to support cancer cell growth, invasion, angiogenesis, and metastasis. These stromal cells have also been recognized as attractive targets to reduce resistance to anticancer therapy and tumor recurrence.^401,402^

**Fig. 3:**
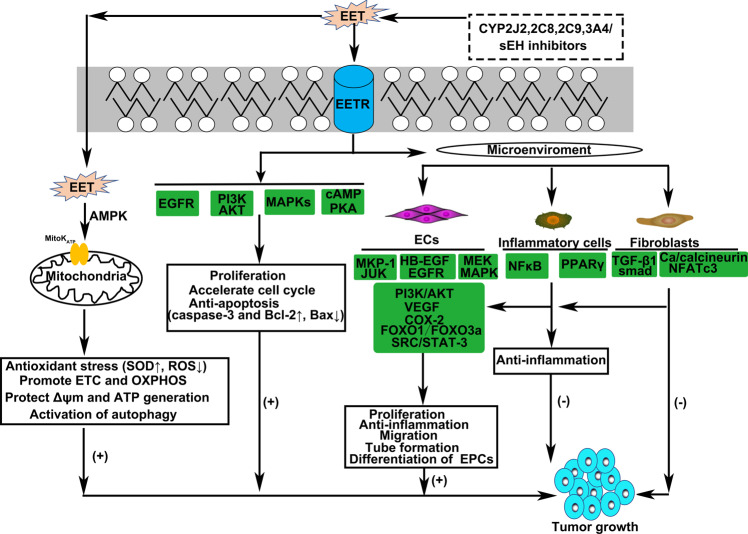
The mechanisms of actions of EETs on tumor growth. EETs accelerated proliferation, cell cycle, and protected carcinoma cells from apoptosis through multiple signal transduction pathways. Besides, EETs improved mitochondrial function and prevented carcinoma cells from oxidative stress damage. In addition, EETs also were found to regulate multiple important cells in TME, such as promoting endothelial cell angiogenesis, fibroblast activation, and anti-inflammation

Inflammatory mediators and inflammatory cells in the inflammatory microenvironment promote the transformation of normal cells to cancer cells in the early stage of cancer, promote the growth and development of cancer cells, and induce tumor immune escape.^367^ An early paper demonstrated that physiological concentrations of EETs or overexpression of CYP2J2 prevented leukocyte adhesion to the vascular wall by a mechanism involving inhibition of transcription factor NF-κB and IκB kinase.^403,404^ Similarly, CYP2J2 transgenic, CYP2C8 transgenic and sEH^-/-^ mice each exhibited a significant attenuation of endotoxin-induced activation of NF-κB signaling, cellular adhesion molecule, chemokine, and cytokine expression, and neutrophil infiltration in vivo.^202^ That is, inhibition of NF-κB is one of the central mediators of the anti-inflammatory response of EETs. NF-κB had been generally recognized as a critical link between chronic inflammation and cancer.^405^ Thus, it is tempting to speculate that the CYP-EET/sEH system in TME could manipulate the activation state of immune cells thus contribution to tumor suppression. However, many CYP enzymes, e.g., CYP2C8 and 9 generate reactive oxygen species as a byproduct of their reaction which can, in turn, stimulate NF-κB. In the vascular system this has been linked with an increased adhesion molecular expression and detrimental effects on vascular function.^406^ Thus, the actions of CYP enzymes on the NF-κB pathway seem to depend on their ability to generate biologically relevant amounts of oxygen-derived free radicals (e.g., CYP2C8 and CYP2C9) while others (e.g., CYP2J2) generate fewer such mediators (Fig. 3).

EETs also increase PPARγ transcription and blocking PPARγ reduces the antiinflammatory effects of the EETs and sEH inhibitors, indicating PPARγ is an effector of EETs.^407^ The infiltrating tumor-associated macrophages are an important additional source of VEGFA, leading to increased vascular permeability and tumor cell metastasis in most solid tumors.^408^ Some monocyte CYP enzymes are differentially expressed in tumor macrophages, one example being CYP2S1 which could be detected in inflamed tissues but was lacking from tumor-associated macrophages in breast cancer metastases.^409^ In addition, CYP4A positive tumor-associated macrophages correlated positively with metastatic niche formation and poor outcome in breast cancer therapy. The inhibition of CYP4A, on the other hand, tended to reduce pre-metastatic niche formation, reflected in a reduced VEGFR-1 positive myeloid cell recruitment.^410^ Apart from macrophages, infiltrating neutrophils also stimulate angiogenesis by secreting VEGFA and other angiogenic growth factors. 14,15-EET was found to induce neutrophil infiltration into dormant metastases to induce a neutrophil reprogramming from a tumor-suppressing to a tumor-promoting phenotype. Depleting neutrophils resulted in the failure of 14,15-EET to promote the development of micro-metastases.^411^

The Hammock laboratory demonstrated that the sEH is a therapeutic target for inflammation for its capacity of inactivating endogenous anti-inflammatory EETs.^412^ In another study, the tobacco smoke-exposed rats treated with a sEH inhibitor resulted in a significant decrease in bronchoalveolar inflammatory cells, including significant reductions in neutrophils, alveolar macrophages, and lymphocytes.^412,413^ sEH inhibition decreases COX-2 protein levels without altering COX-1 expression and decreases inflammatory eicosanoid levels in LPS-challenged mice. The inhibitors also showed synergetic effects with NSAIDs and COX-2 inhibitors in suppressing inflammation.^414^ Thus, sEH inhibitors could be a novel therapeutic method for inflammation-related cancer via their strong anti-inflammation actions especially when combined with COX inhibition, although the pro-angiogenic and tumorigenic products EETs increase.

## AA metabolism in other inflammatory and metabolic diseases

### AA in asthma

Asthma is a chronic disease of the lung caused by airway inflammation and involves airway hyperresponsiveness, mucus overproduction, remodeling, and narrowing of the airway. CysLT1 antagonists, including montelukast, pranlukast, and zafirlukast, are used worldwide for the treatment of asthma. Asthma is mostly associated with type 2 inflammation (The type 2 inflammation is mainly regulated by subpopulations of CD4^+^ T cells known as T helper 2 cells), leading to the release of Th2 cytokines (IL-4, IL-5, and IL-13), IgE production, airway recruitment of eosinophils, and goblet cell metaplasia. It is now understood that some asthmatic inflammation induces the recruitment and activation of Th2 cells and group 2 innate lymphoid cells (ILC2s) by epithelial-derived innate cytokines such as IL-25, IL-33, and thymic stromal lymphopoietin (TSLP).^415,416^ A recent study demonstrated that IL-33 enhanced CysLT1 expression in human peripheral blood lymphocytes (PBLs) in vitro.^417^ LTD4 stimulation induces intracellular calcium mobilization and chemotaxis in PBLs, which express a membrane-bound IL-33-specific receptor, ST2L. Boyce et al. and Doherty et al. independently demonstrated that LTC4 potentiated the activation and migration of ILC2s via CysLT1 or CysLT2 signaling.^304,418,419^ Thus, the CysLT antagonists suppress innate immunological function in asthmatic patients. In addition, LTE4 induces mucin release and submucosal swelling in the nasal mucosa through GPR99 signaling in lung and nasal epithelial cells.^420^ LTE4 stimulation greatly facilitated the chemotaxis of ILC2s differentiated from human PBLs.^421^ LTE4 also enhances the release of Th2 cytokines and pro-inflammatory cytokines (e.g., IL-8 and GM-CSF) from cultured ILC2s in combination with PGD2, IL-25, IL-33, and TSLP.^421^ Thus, in addition to CysLT1 and CysLT2, the LTE4 receptor GPR99 may be a useful therapeutic target for asthma and related diseases such as aspirin-exacerbated respiratory disease.^121,422^

### AA in arthritis

Arthritis is a common inflammatory disease of the joints and includes rheumatoid arthritis (RA) and osteoarthritis. RA is a chronic and systemic inflammatory autoimmune disease that is characterized by inflammatory cell infiltration, synovial hyperplasia, and bone and cartilage destruction.^423^ There are both acute and chronic models of rheumatoid arthritis, the mice expressing both the T cell receptor (TCR) transgene KRN and the MHC class II molecule Ag7 (K/BxN mice) and collagen-induced arthritis (CIA) model, respectively.^424^

NSAIDs are predominantly used for controlling pain and inflammation and are administered as a first‐line medication for newly diagnosed RA cases. Recently, researchers have concentrated mostly on NSAIDs that inhibit COX‐2 selectively. Their pain alleviating and anti‐inflammatory effects are similar to conventional NSAIDs.^425,426^

Luster and Haribabu et al. clearly demonstrate that BLT1 is critically involved in inflammatory arthritis using several RA mouse models, including the K/BxN STA^304,427,428^ and CIA^429^ models. In the K/BxN STA model, the authors demonstrated that BLT1 is required for peripheral neutrophil recruitment into the joint and the resultant induction of IL-1 via immune complex-Fc R interactions. Inflammatory cytokines such as IL-1β and TNFα accelerate the production of the chemokines CXCL1, CXCL5, and CCL5 from fibroblast-like synovial cells, endothelial cells, and macrophages. These chemokines subsequently promote the late phase of neutrophil recruitment by activation of CCR1 and CXCR2, the receptors for CCL5 and CXCL1/5, respectively. Importantly, a BLT1 antagonist, CP105696, improved the incidence of arthritis in both the preventive and therapeutic modes.^304^ Taken together, BLT1 may be a promising therapeutic target for arthritis.

### AA cascades in homeostasis of metabolic diseases

Diabetes mellitus type 1 (DM1) and DM2 are by definition associated with recurrent hyperglycemia due to insufficient insulin production and insulin resistance, respectively. Hyperglycemia induces the production of pro-inflammatory mediators by PMNs, gives rise to oxygen radical formation, hampers PMN chemotaxis, and supports the adhesion of PMNs to the vasculature in diabetic mice.^78,430^ In addition, free fatty acids activate the NLRP3-ASC inflammasome, and a disruption of the associated Nod-like receptors (NLRs) protects against insulin resistance and hyperglycemia in obesity.^431^ Glucose and lipid metabolism share various metabolic pathways. Consequently, disturbances in glucose and lipid metabolism are tightly related, and over-nutrition and/or obesity ensure both, dysregulated lipid metabolism and hyperglycemia. Macrophages derived from diabetic mice have a pro-inflammatory phenotype and express high levels of acyl-CoA synthetase 1 (ACSL1). ACSL1 is implicated in the generation of pro-inflammatory PGs, such as PGE2, thus fostering pro-inflammatory functions of macrophages.^432^ Consequently, disruption of ACSL1 in myeloid cells significantly reduces the inflammatory signaling in diabetic macrophages and attenuates the progression of the atherosclerotic lesions in diabetic mice.^432^ PGE2 inhibits insulin secretion in pancreatic islets, and enhances pancreatic β cell dysfunction and destruction, whereas PGI2 improves the insulin sensitivity of pancreatic cells. Contrarily, PGE2 fosters adipogenesis in white fat tissue and induces glycogenolysis and gluconeogenesis, thus alleviating insulin resistance of adipocytes.^433^ Recently, PGF2, which is synthesized at higher levels in diabetic mice, was linked to hepatic gluconeogenesis, a major driver of fasting hyperglycemia in DM2.^8,434^

12/15-LOX enzymes are linked to DM via the production of various HPETEs, which interact with PPARs, and are implicated in the cytokine-mediated damage of pancreatic cells. It is therefore not surprising that 12/15-LOX knockout mice demonstrate a partial resistance to diabetes development.^433,435–437^ Similarly, LTs, produced by 5-LOX and 12-LOX-derived HETEs, inhibits pancreatic insulin secretion, and the genetic disruption or pharmacological inhibition of these LOXs protects against pancreatic islet cell destruction in diabetic mice.^438^ LTB4 has been found to be essential for the recruitment and activation of adipose tissue B2 lymphocytes, which contribute to the establishment of insulin resistance following a high-fat diet.^439^

CYP-derived EETs and 20-HETE induce insulin secretion and protect pancreatic islet cells from apoptosis.^21,440^ Diabetes and obesity are associated with an enhanced expression of the sEH, and genetic deletion of the sEH ensues an improved insulin sensitivity and an anti-apoptotic effect on pancreas islet cells in the murine diabetes model.^441^ Recent data suggest that CYP enzymes and EETs are involved in the homeostasis of metabolic diseases, including obesity and diabetes.^442,443^ Previous study has also shown that sEH is expressed in adipose tissue,^444^ hepatocytes,^445^, and pancreatic islets. At least in part, it is speculated that EETs play an important role in the treatment of diet-associated metabolic diseases. Our previous study indicated that, in addition to lowering blood pressure, CYP2J3 overexpression improved insulin resistance in rats treated with fructose and in db/db diabetic mice. This improvement in insulin resistance was associated with the activation of insulin receptor signaling and adiponectin-mediated AMPK signaling pathways.^442,446^ CYP2J3 gene delivery markedly reversed insulin resistance via upregulated AMPK signaling, which was associated with decreased ER stress response in adipose tissue.^442^ CYP2J3-derived EETs alleviate insulin resistance, at least in part through upregulated endothelial nitric oxide synthase expression in rats treated with fructose, which was associated with activation of MAPK and protein kinase C signaling pathways. Genetic disruption or pharmacologic inhibition of sEH led to an enhancement of insulin signaling and sensitivity, increased islet size and vasculature, and decreased plasma glucose.^447^ sEH knockout or inhibition not only attenuated insulin resistance in diabetes but also enhanced glucose-stimulated insulin secretion from islet cells and decreased islet cell apoptosis. Interestingly, several studies have shown that the disruption of sEH enhanced islet glucose-stimulated insulin secretion through AMPK signaling and decreased islet cell apoptosis in diabetes.^447^ Inhibiting sEH activity provided significant protection against islet β cell damage and improved glucose homeostasis in streptozotocin-induced diabetes.^447,448^ Moreover, 5,6-EET directly stimulates the release of insulin but has no effect on glucagon release. In contrast, 8,9-EET, 11,12-EET, and 14,15-EET increase glucagon release without affecting insulin secretion.^449^ Accordingly, the therapeutic potential of sEH inhibitors was tested in several clinical trials. Whereas results of some trials are still pending (e.g., NCT03486223), a Phase II trial introducing a thrice-daily application of an orally administered sEH inhibitor in patients with mild to moderate arterial hypertension and pre-diabetes, failed to demonstrate an improvement of insulin sensitivity (NCT00847899).

Finally, AA also facilitates the production of anti-inflammatory LXs. The latter was reported to improve insulin sensitivity and may prevent the development of DM.^450^ For instance, LXA4 inhibits IL-6, TNFα, and ROS production thus hampers obesity-associated inflammation and has an anti-diabetic effect.^451–453^ LXs are endogenously produced eicosanoids with a spectrum of anti-inflammatory, proresolution, and antifibrotic bioactions. Furthermore, LXs stimulate nonphlogistic macrophage phagocytosis of apoptotic neutrophils both in vitro and in vivo, which is also associated with a shift from the release of proinflammatory to anti-inflammatory cytokines.^451,454^

Adipose tissue is a metabolically active endocrine organ, comprising adipocytes and other cells, such as macrophages and preadipocytes. A key factor in the development of adipose inflammation is a switch in the phenotype of the adipose tissue macrophages (ATMs). Tissue macrophages are heterogeneous and display phenotypic plasticity. Classically activated M1 macrophages are described as proinflammatory, whereas alternatively activated M2 macrophages are thought to be proresolving.^451,455^ In lean subjects, ATMs are predominantly of an M2 phenotype. However, factors such as obesity cause adipose hypoxia and hyperglycemia, the latter, e.g., leading to steatosis and hepatic stress responses with the production of proinflammatory mediators, contributing to systemic inflammation. In combination, these factors cause adipose inflammation and recruitment of macrophages, predominantly of an M1 phenotype.^451,456^ M1 ATMs secrete proinflammatory mediators, which further exaggerate inflammatory responses promoting adipose insulin resistance. The subsequent release of free fatty acids results in systemic lipotoxicity, which contributes to the pathology of T2DM. It has previously been shown that macrophage depletion or blocking macrophage recruitment protects mice from adipose inflammation and IR.^457^ Promoting a shift of M1 to M2 phenotype may, however, be a more physiological approach to subverting adipose inflammation, since the macrophages are required for effective resolution. A previous study reported that LXA4 treatment of macrophages subverted macrophage-induced IR and restored glucose uptake in adipocytes. This effect was associated with rescued AKT activation and reduced secretion of proinflammatory cytokines, including TNFα. These data expand the repertoire of bioactions associated with LXA4 and provide initial ex vivo and in vitro evidence to support the potential of using proresolving mediators, such as LXA4, as a therapeutic to reduce adipose inflammation and IR for instance in T2DM.^451^

In summary, AA derivatives play diverse and partly contrasting roles in the pathogenesis of DM. Therefore, research in AA metabolism and its enzymatic pathways may identify novel targets for the treatment of DM and its associated co-morbidities.

## Clinical studies by targeting AA pathway and outlook for novel therapeutic applications

Based on the widely established concept that COX, LOX, and the CYP-EET/sEH axis play important roles in cardiovascular disease as well as in tumor growth and metastasis, the development of drugs or biological products that target COX, LOX, CYP enzymes, or the sEH has bright prospects.

### Clinical trials associated with AA-COX pathway

Since bioactive lipid metabolites from AA metabolism can be potent mediators of inflammation and cancer progression, COX inhibitors act as important mediators of these cellular responses. As known, aspirin acts as a drug against pain and inflammation, has been widely used in many solid cancers, such as lung cancer, colorectal cancer, and esophageal cancer (e.g., NCT02169271, NCT00468910, NCT00474903). In addition, overexpression of COX-2 in several different types of solid tumors has been reported and supported by animal studies that confirmed the association of genetic COX-2 overexpression with tumorigenesis and malignant progression.^458^ Consistently, more and more s clinical trials showed that COX-2 may be an important target in cancer therapies. Various COX-2 inhibitors (e.g., celecoxib, apricoxib) have also been developed against different cancers (e.g., NCT00582660, NCT00466505, NCT01111591, NCT01532362). In addition to cancers, aspirin also has been used as a medicament for antiplatelet aggregation. It is often prescribed by coronary artery disease patients because of its unique ability to permanently prevent platelets from aggregating and forming a blood clot. In addition, more and more COX-2 inhibitors (such as naproxen sodium, etoricoxib and celecoxib) have been introduced in preventing pain and inflammation in arthritis and osteoarthritis (e.g., NCT03699293, NCT00746720, NCT02198924). Specially, the PGI2 derivative, beraprost, has been reported to reduce pulmonary arterial hypertension (PAH) (NCT00990314). Moreover, treprostinil, a DP1 and EP2 agonist and selexipag, an IP receptor agonist, were both newly approved by FDA to treat PAH (e.g., NCT01268553, NCT01106014) (Table 4).

**Table 4:** Clinical trials associated with AA and its metabolites in different diseases or conditions

Date of Registration (y/m)	Registration number	Drug	Diseases or conditions	Target	Phase
*AA and its metabolites in cancers*					
2014/11	NCT02169271	Aspirin	Lung cancer	COX-1/2	2
2007/3	NCT00468910	Aspirin	Colorectal cancer	COX-1/2	2
2007/4	NCT00474903	Aspirin	Esophageal cancer	COX-1/2	2
2001/12	NCT00582660	Celecoxib	Colorectal adenoma	COX-2	2
2005/5	NCT00466505	Celecoxib	Colorectal cancer	COX-2	2
2010/2	NCT01041781	Celecoxib	Lung cancer	COX-2	3
2001/11	NCT00084409	Iloprost	Lung cancer	PGI2	2
2010/4	NCT01111591	Celecoxib	Bile duct cancer, Pancreatic cancer	COX-2	4
2012/2	NCT01532362	Apricoxib	Non-small cell lung cancer	COX-2	NA
2017/3 2003/3	NCT02950480 NCT00056004	Zafirlukast Zileuton	Breast cancer Lung cancer	LTD4 inhibitor 5-LOX inhibitor	2 2
2010/5 2014/1	NCT01021215 NCT02047149	Zileuton Zileuton	Tobacco use disorder Chronic myelogenous leukemia	5-LOX inhibitor 5-LOX inhibitor	1 1
2013/12	NCT02012920	Seviteronel	Castration-resistant prostate cancer	CYP17 inhibitor	2
2015/3	NCT02381080	Ibrutinib	B-cell chronic lymphocytic leukemia	CYP3A inhibitor	1
2014/4	NCT02122770	MLN4924	Advanced solid tumors	CYP3A inhibitors	1
*AA and its metabolites in CVD*					
2004/6	NCT00646906	Aspirin	Myocardial infarction, arthritis	COX-1/2	NA
2009/11	NCT00990314	Beraprost	Pulmonary arterial hypertension	PGI2 derivative	2
2010/8	NCT01268553	Treprostinil	Pulmonary arterial hypertension	DP1 and EP2 agonist	4
2009/12	NCT01106014	Selexipag	Pulmonary arterial hypertension	IP receptor agonist	3
2006/7	NCT00379808	Montelukast	Coronary heart disease	Cys-LT1-receptor antagonist	NA
2006/7	NCT00358826	VIA-2291	Coronary artery disease	5-LOX inhibitor	2
2006/7	NCT00352417	VIA-2291	Atherosclerosis	5-LOX inhibitor	2
2009/9	NCT00872599	Fenofibrate	Hypertension	PPARα activator	4
2018/5	NCT03318783	GSK2256294	Subarachnoid hemorrhage	sEH Inhibitior	1
2006/1	NCT00283335	Gemfibrozil	Coronary heart disease	CYP enzyme inhibitor and PPARα activator	3
2005/4 2009/1	NCT00108511 NCT00847899	Gemfibrozil AR9281	Hypertriglyceridemia Hypertension, impaired glucose tolerance	CYP enzyme inhibitor and PPARα activator sEH Inhibitior	1 2
*AA and metabolites in other diseases*					
2018/9	NCT03699293	Naproxen sodium	Arthritis	COX-1/2	4
2006/5	NCT00746720	Etoricoxib	Osteoarthritis	COX-2	2
2014/12	NCT02198924	Parecoxib and Celecoxib	Pain, inflammation	COX-2	4
2017/10	NCT03163966	CR6086	Rheumatoid arthritis	EP4 Antagonist	2
2002/4	NCT00092105	Montelukast	Asthma	Cys-LT1-receptor antagonist	3
2006/6	NCT00461032	Montelukast	Asthma	Cys-LT1-receptor antagonist	3
2010/6	NCT01147744	GSK2190915	Asthma	FLAP inhibitor	2
2008/7	NCT00723021	Zileuton	Asthma	5-LOX inhibitor	2
2020/5	NCT03486223	GSK2256294	Diabetes mellitus, endocrine system diseases, obesity	sEH Inhibitior	2
2009/1	NCT00847899	AR9281	Hypertension impaired glucose tolerance	sEH Inhibitior	2
2019/7	NCT04075110	Montelukast	Obesity; endocrine; T2DM	Cys-LT1-receptor antagonist	1
2015/4	NCT02291666	CRCHUM-MT cocktail	T2DM	CYP450	4

### Clinical trials associated with AA-LOX pathway

Recent studies showed that a 5-LOX inhibitor, VIA-2291, possessed a protective role against coronary artery disease (NCT00358826) and atherosclerosis (NCT00352417). Recently, another specific 5-LOX inhibitor, zileuton, usually used to modify airway inflammation (NCT00723021), was also found to prevent tumor growth (NCT00056004 and NCT02047149). Montelukast, an effective drug against asthma, was also found to prevent coronary artery disease by targeting Cys-LT1-receptor (NCT00379808). Besides, in obesity or T2DM, montelukast may have a role in regulating homeostasis of metabolic diseases (NCT04075110) (Table 4). Despite these promising effects in both asthma and in CVD montelukast may lead to severe neurospyschiatric problems. The biologic mechanisms underlying the neuropsychiatric events are not well understood, but evidence from animal studies suggests that montelukast could act directly on cells in the brain. Orally administered montelukast (10 mg/kg/day, 7 days) was detectable in brain tissue and cerebrospinal fluid (CSF) in rats,^459^ providing evidence for its ability to cross the blood-brain barrier. Montelukast is a potent competitive antagonist (IC50 = 2.3 nM) at its target, the CysLT1 receptor.^460^ However, expression of the CysLT1R in the normal human brain is very low/non-existent, implying that the compound may have off-tartet effets Montelukast is also a competitive antagonist of (IC50 = ~60 nM) of GPR17, a G-protein-coupled receptor, which is expressed on neurons and glial cells in the human brain.^461^ GPR17 is recognized as a regulator of oligodendrocyte development and remyelinating function.^462^ Montelukast inhibition of GPR17 function on neurons and/or glial cells may contribute to the biologic processes underlying the observed neuropsychiatric events associated with montelukast treatment.

### CYP epoxygenases inhibitors and EET antagonists

There are no clinical trials that directly targeting CYP enzymes or their direct products. Thus, below, we will mainly introduce the CYP inhibitors and EET antagonists, which may have the potential to be used in the future.

Both CYP epoxygenases inhibitors and EET antagonists are effective approaches to reduce EETs production and their biological effects. Two fatty acid derivatives [2-(2-propynyloxy)-benzenehexanoic acid (PPOH) and its metabolically stable congener N-(methylsulfonyl)-2-(2-propynyloxy)-benzenehexanamide (MS-PPOH)] are generally used as specific EETS synthesis inhibitors.^463^ The former compound shows wide inhibition on CYP2B and 2C epoxygenases while MS-PPOH prefers to inhibit CYP2C9 and CYP2C11 subtypes.^464^ The lipid-lowering drug gemfibrozil also shows widespread inhibition on CYP epoxygenases including CYP2C8 with a Ki range between 9.3 and 270 mM, CYP2C9 and CYP2C19 with Ki values of 5.8 and 24 mM, respectively, and CYP1A2 with a Ki of 82 mM.^465^ In vitro study, MS-PPOH abolished migration and tube formation of endothelial cells exposed to hypoxia or CYP2C9 overexpression. In addition, blocking EET synthesis by MS-PPOH] impaired the ability of prostate carcinoma cells (PC-3, DU-145, and LNCaP) to invade and migrate.^399^ In both primary and secondary prevention studies, gemfibrozil reduced cardiovascular endpoints and coronary disease mortality.^466,467^ A number of recent studies reveal that apart from its lipid-lowering effects, gemfibrozil can also regulate many other signaling pathways responsible for inflammation, switching of T-helper cells, cell-to-cell contact, migration, and oxidative stress.^468^ In addition, another epoxygenase inhibitor 17-ODYA had also been found to inhibit the proliferation, migration, invasion, and adhesion in solid cancer cells^262^ and multiple myeloma cells,^469^ and accelerate cancer cell apoptosis induced by TNFα.^261^ Human umbilical vein endothelial cell proliferation and tube formation are also restrained by 17-ODYA treatment with an associated reduction in EET production.^212,470^ In addition, CYP3A4, another epoxygenase responsible for EET production, was highly expressed in breast cancer and associated with breast cancer development and progression.^471^ Treatment of breast cancer cells with ketoconazole and azamulin, selective inhibitors of CYP3A4, inhibited cell proliferation and conferred sensitivity to the selective estrogen receptor modulator 4-hydroxytamoxifen.^472^ Thus, CYP epoxygenases inhibitors are expected to be potential drugs against tumor growth and metastasis via endothelium-dependent and independent mechanisms. Although various CYP epoxygenases inhibitors (e.g., SKF525A, clotrimazole) had been synthesized,^473^ few inhibitors enter into clinical trials as an anticancer therapy.^474^ Firstly, these inhibitors often target multiple CYP homologous genes, resulting in changes of various lipid metabolites. Secondly, inhibiting CYP epoxygenase pathway may be followed by an increase of other arms of the eicosanoid pathways, such as COX or LOX activity, resulting in the generation of metabolites with angiogenic and tumorigenic potential. In addition, CYP inhibition influenced the bioavailability of anticancer agents such as paclitaxel and docetaxel, vinorelbine, and tamoxifen,^475^ limiting their clinical promotion.

As for EET-receptor antagonist, it’s a pity that so far, the EET receptor has not been conclusively identified despite numerous evidence linking the presumptive receptor to a GPCR. Therefore, developing compounds specifically binding to the uncertain EET receptor seems to be impractical. Interestingly, the synthetic 14,15-EET analogues, such as 14,15-EEZE, 14,15-epoxyeicosa-5(Z)-enoic acid 2-[2-(3-hydroxypropoxy)-ethoxy]-ethyl ester [14,15-EEZE-PEG] and 14,15-epoxyeicosa-5(Z)-enoic-methylsulfonylimide [14,15-EEZE-mSI], competitively suppressed the effects of EETs and are identified as EET-specific antagonists.^399^ Cancer cells treated with synthetic EET antagonists prevented EET-induced cell invasion and migration in vitro.^399^ In addition, 14,15-EEZE significantly inhibited migration^392^ and proliferation of CYP3A4 enhanced tumor cells and endothelial cells overexpressing CYP2C9.^472^ Consistent with these in vitro findings, mice treated with EET antagonists showed reduced primary tumor growth and multi-organ metastatic potential.^400^

The sEH inhibitors, stabilizing endogenous EETs, are promising drug candidates for multiple human diseases. In prophase animal models, various pharmacological sEHIs, such as AUDA, AUDA-BE, t-AUCB, TPPU, and 1-adamantan-1-yl-3-urea (AEPU), showed that they are able to effectively lower hypertension,^476^ alleviate multi-organ inflammation^477,478^ and neuropathic pain,^413,479^ inhibit cardiac hypertrophy,^480^ detrimental cardiac remodelings and HF,^176^ as well as to attenuate hepatocellular necrosis and hepatic fibrosis^481^ and renal interstitial fibrosis and inflammation.^482^

Given the pro-angiogenic and pro-tumorigenic action of EETs, reduction of EET synthesis may provide clinical benefit for cancer patients. Many researchers emphasized that the anti-tumor and anti-metastatic roles of PPARα activation depended on the suppression of endothelial function.^483–485^ A study conducted by Pozzi et al.^486^ pointed out that the anti-tumorigenic and anti-angiogenic properties of PPARα are AA epoxygenase-mediated. Treatment with PPARα ligands such as Wy-14643 or fibrates downregulates CYP2C9 and CYP2C44 expression in human and murine endothelial cells, respectively, and reduces EET biosynthesis.^484,486^ In a mouse xenograft model of tumorigenesis, disruption of host CYP2C44 epoxygenase suppressed tumor growth and vascularization and abrogated the anti-tumor effects of PPARα agonists.^486^ In addition, Mice treated with PPARα ligands also show reduced primary and metastatic non-small cell lung cancer (NSCLC) tumor growth, tumor angiogenesis, endothelial CYP2C44 expression, and circulating EET levels.^487^ Taken together, these results indicate that activation of PPARα and consequent downregulation of CYP2C expression may be a promising anti-cancer approach.

Besides PPAR, other nuclear receptors, including the aryl hydrocarbon receptor (AhR), constitutive androstane receptor (CAR), pregnane X receptor (PXR), and glucocorticoid receptor (GR), were noted to participate in receptor-dependent mechanisms of CYP induction,^488,489^ where they directly bind to their response DNA sequences to regulate CYP gene expression. Genetic studies indicated that the AhR forms heterodimers with AhR nuclear translocator (ARNT), and then binds to xenobiotic response elements in promoter regions of CYP^490^ 18686044. CAR targets and regulates CYP3A4, CYP2C8, and CYP2C9 in response to phenobarbital treatment.^491,492^ In human hepatocytes, PXR activates CYP3A genes in response to diverse chemicals, including certain natural and synthetic steroids, steroid metabolites, and several clinical drugs. In addition, PXR also activates other CYP genes including members of the 2B and 2C families.^493^ PXR, CAR, and PPAR are orphan receptors, which belong to the nuclear receptor/steroid receptor superfamily, play transcriptional regulatory roles via forming heterodimerize with the retinoid X receptor (RXR) after activation in the nucleus. Expression of PXR, CAR, and RXR are under transcriptional control of the GR. Therefore, the expression of CYP genes may be controlled by a cascade of signal transmissions: GR-[PXR/CAR/RXR]-CYP. In addition, hepatocyte nuclear factor 4α (HNF4α) and other members of liver-enriched transcription factors, including HNF1α, HNF2α, CCAAT/enhancer-binding protein α (C/EBPα), HNF3γ (FOXA), and HNF6, have been shown to regulate the constitutive expression of CYP2C genes.^494,495^ This extensive regulatory network provides the potential for the development of drugs targeted at inhibiting CYP-EETs.

### MicroRNA-mediated regulation of CYP epoxygenases and tumor therapy

MicroRNAs (miRNA) are short non-coding RNA molecules of 21–23 nucleotides that modulate the stability and/or the translational efficiency of target messenger RNAs.^496^ Several miRNAs had been associated with the regulation of CYP epoxygenases function, which presents a novel and attractive avenue for cancer therapy.^497^ Chen et al.^498^ found the expression level of CYP2J2 was inversely proportional to that of let-7b in lung squamous cell cancer tissues and further uncovered that let-7b diminished cell proliferation and promoted apoptosis of tumor cells via posttranscriptional repression of CYP2J2. In addition, the upregulation of miR-128-3p is inversely correlated with the expression of CYP2C9 in hepatocellular carcinoma tissues. MiR-128-3p is able to suppress CYP2C9 expression/production in human hepatic cells by specifically targeting the 3’-UTR of CYP2C9 mRNA molecules.^499^ The expression of CYP2C9 is also reported to be directly and negatively regulated by miR-130b.^500^ The translation efficiency (protein/mRNA ratio) for CYP2C8 was significantly inhibited by miR-103 and miR-107, which also targeted CYP2C9 and CYP2C19 to a lesser degree than CYP2C8 in the human Liver.^501^ Taken together, miRNAs-mediated regulation of CYP epoxygenases may contribute to cancer treatment. Especially, various nanoparticles are being developed and employed to load microRNAs, overcoming challenges associated with microRNA degradation, transient expression and poor targeting.^502^

### The potential risks of drugs targeting AA pathway for human application

Although drugs targeted at AA metabolism exhibited multiple therapeutic effects on CAD and cancer, their possible side effects deserve mention here. The gastrointestinal (GI) side effects are ranked as the most common among NSAIDs-related adverse events. However, COX-2 selective NSAID agents seem to reduce GI side effects compared with traditional non-selective drugs.^503^ In addition, another major concern across all forms of NSAID therapy is the cardiovascular side effects. Except for aspirin, other drugs in the NSAIDs class are associated with increased risk of cardiovascular side effects including hypertension, stroke, heart attacks, and HF.^504^ Up to now, zileuton is the only approved 5-LOX inhibitor but it has numerous disadvantages, such as hepatic toxicity and adverse pharmacokinetic profile derived from a short half-life.^505^ Masferrer et al.^506^, demonstrated the inhibitory potency of PF-4191834 on LTB4 production by use of rat air pouch model. PF-4191834 has also completed phase II (NCT00723021) clinical trial for asthma but phase II for knee osteoarthritis was terminated due to a serious adverse event (NCT01147458) such as syncope, acute hepatitis, and gastric ulcer hemorrhage. Little clinical data regarding the safety of 12/15 LOX inhibitors can be referenced, because their side effects usually preclude them from entering into routine clinical use.^507^ Antagonists against cysteinyl leukotriene receptor (CysLTR) type 1, including montelukast, pranlukast, and zafirlukast, has been linked to apparent liver injury, various neuropsychiatric events^508^ and skin adverse reactions.^509^ CYP inhibitors seem be well tolerated and tested with low risk. On the one hand, numerous substances in nature and many long-used drugs in clinical practice are non-selective inhibitors of CYP. On the other hand, the activation of the compensatory pathway makes it possible for another pathway to enhance compensation when one CYP enzyme is inhibited. For example, type 1 angiotensin-II receptor antagonist telmisartan used as an antihypertensive drug and H1 receptor antagonists, terfenadine used as antiallergic agent for many years, have been identified as potent CYP2J2 inhibitor at concentrations that are reached during clinical use and are well tolerated by patients,^510,511^ because terfenadone strongly inhibited CYP2J2-mediated metabolism process.^512^ However, the side effects of CYP inhibitors can not be ignored because of the important effects of CYP enzyme on drug metabolism and their double-edged sword on CVD and cancer treatment. No sEH inhibitor has been presented to the market yet. Only some hopeful sEH inhibitor candidates are subjected now to clinical trials such as GSK2256294A in Phase-I and AR9281 in Phase-II against chronic obstructive pulmonary disease (COPD) and hypertension.^513,514^ However, the possibility of angiogenic effects when inhibiting sEH needs to be further evaluated.

## Conclusion and future direction

Considerable data indicate that COX, LOX, CYP enzymes, and their metabolites of AA play important roles in the initiation and development of human diseases, especially cardiovascular and cancer. Although the specific mechanisms are not entirely clear, increasing evidence indicates that the CYP pathway has potential as a therapeutic target in these two disease areas. An important challenge for future research will be to obtain a better understanding of the different biological activities of AA metabolites such as EETs generated by the CYP/sEH axis serve both endogenous cardiovascular protectors and promotor of cancers. Ultimately, understanding the basic cellular mechanisms of these metabolites will enhance our knowledge and lead to better management of CVD and cancer and well as inflammatory diseases via developing novel drugs in key point of AA metabolism pathways.

## Supplementary information

Marked version of revised manuscript
